# Cost-effectiveness analysis of genotype-guided optimization of major depression treatment in Qatar

**DOI:** 10.1080/20523211.2024.2410197

**Published:** 2024-10-25

**Authors:** Dina Abushanab, Shaban Mohammed, Rania Abdel-latif, Wadha Al-Muftah, Said I. Ismail, Moza Al Hail, Wafa Al-Marridi, Oraib Abdallah, Noriya Al-Khuzaei, Asma Al-Thani, Daoud Al-Badriyeh

**Affiliations:** a Pharmacy Department, Hamad Medical Corporation, Doha, Qatar; b Qatar Genome Program, Qatar Precision Health Institute, Qatar Foundation, Doha, Qatar; c College of Medicine, QU Health, Qatar University, Doha, Qatar; d Pharmacy Department, Mental Health Services, Hamad Medical Corporation, Doha, Qatar; e Medical and Health Sciences Office, Qatar University, Doha, Qatar; f College of Pharmacy, QU Health, Qatar University, Doha, Qatar

**Keywords:** Pharmacogenetics, antidepressive agents, depressive disorder, cost-effectiveness analysis, quality-adjusted life years

## Abstract

**Background:**

Pharmacogenetic testing improves the efficacy and safety of antidepressant pharmacotherapy for moderate-severe major depressive disorder by identifying genetic variations that influence medication metabolism, and adjusting treatment regimens accordingly. This study aims to assess the cost-effectiveness of implementing a pharmacogenetic testing approach to guide the prescription of antidepressants.

**Methods:**

From the public hospital perspective, we developed a two-stage decision tree diagram of a short-term 6-week follow up, and a lifetime Markov model with 3-month cycles. The analysis compared the current standard of care with the alternative strategy of Pharmacogenetic-guided (multi-gene panel) testing in adult patients with moderate-severe major depressive disorder. Clinical outcomes and utilities were obtained from published studies, while healthcare costs were locally available. The short-term incremental cost-effectiveness ratio was against treatment response without side effects and without relapse, and against treatment response with/without side effects and without relapse. The long-term incremental cost-effectiveness ratio was against the quality-adjusted life year gained and years of life saved.

**Results:**

Adopting the pharmacogenetic-guided therapy for adult patients with moderate-severe major depressive disorder in Qatar resulted in cost savings of Qatari Riyal 2,289 (95% confidence interval, −22,654–26,340) for the health system. In the short term, the pharmacogenetic-guided testing was associated with higher response rates without side effects and without relapse (mean difference 0.10, 95% confidence interval 0.09–0.15) and higher response rates with or without side effects and without relapse (mean difference 0.05, 95% confidence interval 0.04–0.06). For long term, the pharmacogenetic-guided testing resulted in 0.13 years of life saved and 0.06 quality-adjusted life year gained, per person, along with cost savings of Qatari Riyal 46,215 (95% confidence interval-15,744–101,758). The sensitivity analyses confirmed the robustness of the model results.

**Conclusion:**

Implementing pharmacogenetic testing to guide antidepressant use was found to improve population health outcomes, while also significantly reducing health system costs.

## Key points

- The genotype of patients with depression plays a role in the response of patients to antidepressant therapies.
- Optimising depression therapy through pharmacogenetics testing (PGx) improves response and reduces adverse effects.
- We undertook the first cost-effectiveness analysis of the implementation of a PGx-guided depression treatment approach compared to the standard of care (SoC) in Qatar.
- The PGx testing to guide antidepressant use improved patient health outcomes at a reduced overall cost of patient management.

## Background

Major depressive disorder (MDD) is a significant global health concern, recognised as the third leading cause of disease burden in 2008 and projected to become the foremost cause by 2030 (World Health Organization, Executive Board, 2011). Diagnosing MDD requires a persistently low mood or anhedonia, accompanied by four additional symptoms such as feelings of guilt, fatigue, and suicidal thoughts (Tolentino & Schmidt, 2018). This complexity is compounded by biological, genetic, environmental, and psychosocial factors, as well as emerging theories that emphasise intricate neuroregulatory systems rather than solely focusing on neurotransmitter imbalances (Bains & Abdijadid, 2024; Remes et al., 2021). Moreover, individuals suffering from MDD are at an increased risk for comorbid anxiety and substance use disorders, which can further exacerbate the risk of suicide. Additionally, MDD can worsen existing medical conditions like diabetes and hypertension, leading to self-destructive behaviours when left untreated (Bains & Abdijadid, 2024).

Globally, MDD affects approximately 5% of the adult population (Bains & Abdijadid, 2024; World Health Organization, Depressive Disorder, 2023), with women being nearly twice as likely to be affected as men (Abate, 2013; World Health Organization, Depressive Disorder, 2023). The prevalence of MDD is particularly common among individuals with limited social connections and those who are divorced, separated, or widowed (Pan et al., 2022). In the context of the Middle East and North Africa (MENA) region, data from the Global Burden of Disease 2019 database indicates that Palestine had the highest rates of prevalence in 2019, with 6,199 cases per 100,000 people, alongside an incidence rate of 7,864 (Moradinazar et al., 2022). Of note, from 1990 to 2019, the rates of depressive disorders have risen in the region, with Saudi Arabia showing the most significant increases (Moradinazar et al., 2022).

Focusing on Qatar, a study conducted in 2017 examined the prevalence of subthreshold depression (SUBDE) and major depressive episode (MDE) among both migrants and non-migrants. A telephone survey involving 2,424 participants revealed an overall depression prevalence ranging from 4.2% to 6.6%, with SUBDE at 5.5% and MDE at 3.6%. The findings also indicated that SUBDE was significantly more common among low-and high-income migrants compared to non-migrants, with Arab ethnicity being associated with higher rates of both SUBDE and MDE (Khaled, 2019).

When it comes to managing MDD, a variety of treatment options are available, such as medications, psychotherapy, interventional therapies, and lifestyle changes (Lam et al., 2024). Initial treatment often involves pharmacological approaches or psychotherapy, with combination therapy generally yielding the best outcomes. The Food and Drug Administration has approved several classes of medications for MDD, including selective serotonin reuptake inhibitors (SSRIs), like fluoxetine and sertraline, which are favoured for their manageable adverse effects. Serotonin–norepinephrine reuptake inhibitors (SNRIs), such as venlafaxine and duloxetine, are prescribed for individuals with comorbid pain disorders. Other options include atypical antidepressants and tricyclics, with the latter being used less frequently due to their side effects. Psychotherapy approaches, including cognitive–behavioral therapy and interpersonal therapy, have also proven effective. For cases resistant to standard therapies, electroconvulsive therapy (ECT), transcranial magnetic stimulation (TMS), and vagus nerve stimulation (VNS) may be considered.

In Qatar, a study on antidepressant prescribing patterns revealed that SSRIs comprised 46% of prescriptions, with escitalopram being the most common at 26% (Bastaki et al., 2021). This highlights the prevalence of SSRIs in clinical practice, aligning with global trends in MDD management.

As the field of mental health evolves, pharmacogenomic (PGx) testing is emerging as a transformative tool, enabling the personalisation of antidepressant prescriptions based on genetic profiles (Bousman et al., 2023; Malsagova et al., 2020). By optimising medication effectiveness and minimising adverse effects, PGx testing can significantly decrease the time required to identify effective therapies, which traditionally spans 4 to 6 weeks. This personalised approach not only improves patient outcomes but also has the potential to be cost-effective by reducing trial-and-error prescribing and reducing long-term healthcare costs associated with untreated or poorly managed MDD.

In Qatar, a robust national healthcare system serves both its citizens and the significant expatriate workforce, offering universal coverage primarily funded by the government. Managed by the Primary Health Care Corporation (PHCC) and Hamad Medical Corporation (HMC), alongside private healthcare options, this system provides comprehensive healthcare services, many of which are free or heavily subsidised. The annual healthcare expenditure is approximately US$6 billion, accounting for 79.1% of government spending (GCC Healthcare Industry, Alpen Capital, 2023).

Recognising the potential of PGx in Qatar, the Qatar Genome Program (QGP) has launched the Qatar Pharmacogenetics Clinical Applications and Research Enhancement Strategies (QPGx-CARES) initiative to facilitate the national adoption of PGx testing (Abdel-Latif et al., 2024). This programme aims to optimise PGx research and clinical applications, ensuring that healthcare providers are equipped with the necessary insights for informed prescribing decisions. As Qatar transitions toward a model of precision medicine, the effective implementation of PGx testing represents a critical step in enhancing mental health care and improving treatment efficacy, while also addressing cost-effectiveness.

Integrating PGx evaluations into standard treatment protocols can optimise medication use, particularly in a diverse population like Qatar, where healthcare resources are limited. By demonstrating the cost-effectiveness of PGx-guided treatments, healthcare policymakers can make evidence-based decisions that not only improve patient care but also enhance the overall efficiency of the Qatari healthcare system (Abdel-Latif et al., 2024).

Therefore, this study aims to provide evidence-based recommendations for healthcare policymakers in Qatar to enhance mental health care strategies, particularly by integrating PGx testing into standard treatment protocols. The primary objective is to evaluate the cost-effectiveness of PGx-guided strategy compared to standard of care (SoC) in managing MDD from the Qatari healthcare system. Specifically, the study seeks to assess the total direct healthcare costs associated with PGx-guided strategy versus SoC, identifying potential cost savings of implementing PGx testing in clinical practice.

By achieving this objective, this study will contribute to the growing body of knowledge on PGx testing in mental health, particularly in the MENA region, as well as support the transition toward personalised medicine. The findings will also inform healthcare practices and policy decisions, ultimately improving patient outcomes and optimising resource allocation within Qatar's healthcare system.

## Methods

### Study design

**Figure 1.**
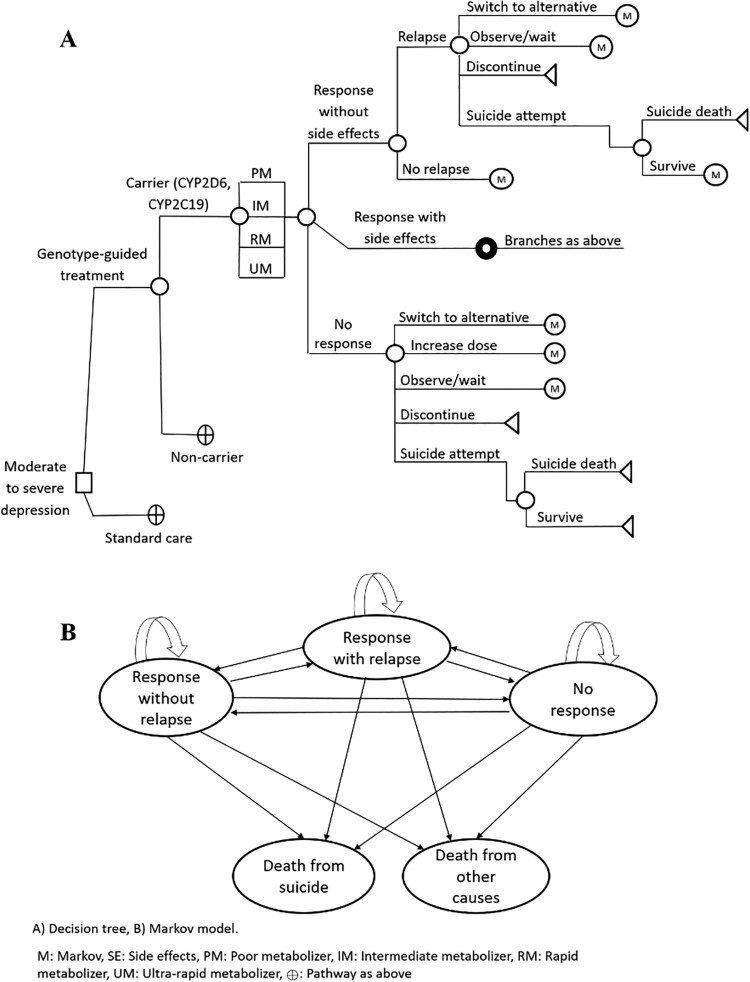
Study economic model.

A model was constructed in Microsoft Excel (Microsoft office) to analyse the cost-effectiveness of using a PGx-guided approach to screen for CYP2C19 and CYP2D6 variants (multi-gene panel testing) in patients with MDD, compared to using the SoC. The model was developed based on the guidelines of the Clinical Pharmacogenetics Implementation Consortium (CIPC) (Bousman et al., 2023), which is to be used in HMC upon PGx implantation, and the clinical expertise of psychiatrists. It consisted of two parts: a decision tree diagram covering a short-term 6-week follow up, followed by a long-term Markov model of 3-month cycles (Figure 1).

### Short-term decision-analytic model

After reviewing previously published studies on CYP2D6 and CYP2C19 genetic testing in patients with MDD (Carta et al., 2022; Fabbri et al., 2021; Shams et al., 2006; Sim. et al., 2015; Sluiter et al., 2019), a 6-week decision-analytic simulation model was developed to assess the use of PGx-guided testing for CYP2D6 and CYP2C19 variants in MDD patients, compared to the current SoC and its potential consequences. The decision tree in the model represented the timeframe from testing for CYP2D6 or CYP2C19 mutations to the initiation of antidepressant treatment with a personalised dose. Patients who underwent PGx-guided testing were differentiated based on whether they were carriers or non-carriers of the alleles. The model considered 18 distinct outcomes of interest, depending on whether patients carried CYP2D6 or CYP2C19 mutations and on the metabolizer class, i.e. poor metabolizer (PM), intermediate metabolizer (IM), rapid metabolizer (RM), or ultra-rapid metabolizer (URM). The probabilities of being assigned to each branch were calculated based on the prevalence of being PM, IM, RM, or URM in the CYP2D6 and CYP2C19 mutations among the Qatari population (Jithesh et al., 2022).

With the PGx-guided strategy, the model assumed a 100% diagnostic rate for patients with detectable CYP2D6 or CYP2C19 mutations using current testing technologies. Thus, test sensitivity and specificity were assumed to be 100%. The analytical validity, which refers to the ability of the test to differentiate between carriers of different alleles, is highly reliable, with a general known value of >99% for CYP enzymes (Daly et al., 2007). Therefore, our model assumed an analytical validity of 100%. In the SoC cohort, patients were directly assigned to receive the standard dose of antidepressants without undergoing PGx-guided testing. The model conservatively assumed that no patients would experience delays in testing. Consequently, no patients in the decision tree model were expected to be affected by declines in response, and the probability of test delay was set at 0.

Patients were differentiated into having ‘success’ or ‘failure’ outcome. ‘Success’ was defined as (i) experiencing a response without side effects, with no relapse within 6 weeks of starting antidepressant treatment, or (ii) experiencing a response with or without side effects, with no relapse within 6 weeks of starting antidepressant treatment. ‘Failure’ was defined as (i) experiencing a response without side effects, with subsequent relapse, (ii) experiencing a response with side effects, with subsequent relapse, or (iii) not experiencing a response within 6 weeks of starting treatment. The side effects of interest were those reported in the literature and expected, as per Qatar practice guidelines, to significantly impact the overall management costs.

In the case of relapse, patients were further categorised into ‘switching to alternative treatment’, ‘observation/waiting’, ‘discontinuation of therapy’, or ‘suicide attempt’. When patients did not achieve a response, they were further categorised into ‘switching to alternative treatment’, ‘titrating the dose’, ‘observation/waiting’, ‘discontinuation of therapy’, or ‘suicide attempt’. In case of ‘switching to alternative treatment’, the patient received an alternative treatment regimen for a duration of 6 weeks, which was assumed to be successful. The ‘titrating the dose’ case reflects dose adjustment rates, ranging from 50% to 100%. The ‘observation/waiting’ implies no changes in the treatment course. In the event of ‘discontinuation of therapy’, the patient received the initial therapy for half of the follow-up cycle (3 weeks), before receiving an alternative treatment regimen for a full 6-week cycle, which was assumed to be successful. For the ‘suicide attempt’, patients were exclusively differentiated into suicidal death or survival. In the case of the failed suicide attempt, a switch to a different treatment was assumed.

For patients who did not achieve a response, which refers to a lack of improvement in depressive symptoms following the administration of an antidepressant medication, they were further categorised into ‘switching to alternative treatment’, ‘titrating the dose’, ‘observation/waiting’, ‘discontinuation of therapy’, or ‘suicide attempt’. In the case of a suicide attempt, the outcome was further differentiated into either a suicide death or survival outcome.

For each treatment strategy, the model considered the probabilities of 18 different pathways, incorporating their respective costs, to calculate the total strategy cost (Figure 1.A). Importantly, the value of an outcome was sum of all values of the outcome throughout all metabolizer classes.

*The overall structure of the decision tree:*

- Decision node: Patients are categorised into two groups:
  - PGx-guided strategy: This involves multi-gene panel testing for CYP2D6 and CYP2C19 variants. Patients are treated based on their specific metabolizer classification, which guides the choice of antidepressant and dosing.
  - SoC: Patients receive standard doses of antidepressants without any PGx testing, with outcomes based solely on traditional treatment methods.
- Chance nodes: In the PGx-guided strategy, patients are further classified based on their metabolizer status into PM, IM, RM, or URM. Each chance node leads to various treatment outcomes, reflecting the likelihood of treatment success or failure based on the genetic profile.
- Endpoints: The model assesses the following endpoints:
  - Success: This is characterised by a response without side effects, with no relapse within 6 weeks of starting antidepressant treatment; or experiencing a response with or without side effects, with no relapse within 6 weeks.
  - Failure: This is characterised by a response without side effects, followed by relapse; experiencing a response with side effects, followed by relapse; or not experiencing a response within 6 weeks of starting treatment.

### Study population

The model was hypothetically constructed to target treatment-naive patients with MDD who scored 20 or higher on the 17-item Hamilton Rating Scale for Depression (HAM-D17), as described in the study by Bradley et al. (2018). We assumed a hypothetical cohort of Qatari patients (*N* = 15,000) affected by MDD, with the demographic and clinical features as reported in the Bradley et al. study (Bradley et al., 2018). The Bradley et al. study is the largest clinical trial of PGx testing conducted to date, involving 20 independent clinical sites. The study utilised the IDgenetix (IDGx) test, which employs a genetic variant panel of 10 genes and incorporates information about concomitant medications. The test provides medication management recommendations based on gene-drug and drug–drug interactions for over 40 medications used in the treatment of depression. The mean age of the study participants was 48 years, and the majority were females. These findings are in line with a local study conducted in Qatar, including that females are at a higher risk of depression compared to males (Bener et al., 2012).

### Perspective

The cost-effectiveness analysis was conducted from a healthcare perspective (i.e. the Mental Health Hospital) (Mental Health Service. Hamad Medical Corporation, 2024), which serves as the primary provider of specialised mental health care and treatment in Qatar. The site is equipped with 145 beds.

### CYP2D6 and CYP2C19 mutation prevalence

The prevalence of mutations was based on Qatari data, which reported the population prevalence of CYP2D6 and CYP2C19 mutations (Jithesh et al., 2022). As per metabolizer class, the mutation prevalence for CYP2D6 is 1.89% for PM, 23.28% for IM, and 8.55% for URM. For CYP2C19, the mutation prevalence is 1.87% for PM, 19.8% for IM, 6.53% for URM, and 29.84% for RM (Jithesh et al., 2022).

### Dosing regimen

Dose adaptations were based on the recommendations from the CPIC guidelines (Bousman et al., 2023). In the PGx-guided strategy, it was assumed that the dosages and treatment for each patient were optimised at the start of treatment, considering their genetic profile and corresponding CYP2D6 or CYP2C19 activity. Depending on their genotypes (PM, IM, RM, or URM), patients in the PGx-guided cohort received different initial dosages. The PM and IM carriers of the variants were classified as high risk, with a predicted CYP2D6 and CYP2C19 enzyme activity of 50%. Therefore, the model assumed that these patients would receive 50% of the standard antidepressant dose. In contrast, the RM, URM, and normal carriers were assumed to receive 100% of the standard dose. In the SoC, patients with different metabolizer profiles received the standard care dose.

### Long-term Markov model

Following the short-term decision-analytic tree, a Markov model was developed to predict long-term outcomes for patients with MDD using 3-month cycles over a lifetime horizon. Throughout the Markov state-transition probability analysis model, patients were assigned to five mutually exclusive health states: ‘response without relapse, with or without side effects’, ‘relapse with or without side effects’, ‘no response’, ‘suicide death’, and ‘death from non-suicide causes’, for both PGx-guided and SoC (Figure 1.B). The model assumed that all patients would initially enter the ‘response without relapse, with or without side effects’ health state. From this health state, patients could transition to the ‘relapse with or without side effects’ state, the ‘no response’ state, or experience death due to suicide or non-suicide causes. The population of interest consisted of patients with MDD aged 48–81 years. The starting age of the population in this Markov model was set at 48 years, which was based on the mean age reported in the Bradley et al. study (2018). Considering the chronic and recurrent nature of MDD, and in order to capture both early and long-term costs and savings associated with the management of MDD, a lifetime time horizon was deemed appropriate. The end age was set at 81 years old, which approximates the life expectancy in Qatar (i.e. 80.88 years in 2024) (Qatar Life Expectancy 1950–2024, 2024). The model tracked patients until death or until they reached 81 years of age in the model, whichever came first.

### Model inputs

For the model parameters, a comprehensive literature search was conducted and, when necessary, assumptions were made as discussed with clinical experts. While there is limited evidence that stratifies treatment outcomes by CYP2D6 or CYP2C19 metabolizer classes, our model focused on the SSRIs, SNRIs, and serotonin modulator and stimulators (SMS) antidepressants that had the strongest associated effects with CYP2D6 or CYP2C19. According to CIPC 2023 (Bousman et al., 2023), paroxetine, vortioxetine, fluvoxamine, and venlafaxine were strongly associated with CYP2D6, while escitalopram and sertraline showed the strongest effect with CYP2C19.

#### Short-term decision-analytic model

The input data for the cost-effectiveness analysis were primarily obtained from Rush et al. (2006), Sim et al. (2015), Bradley et al. (2018), Sluiter et al. (2019), Carta et al. (2022), Fabbri et al. (2021), and Shams et al. (2006). The weighted probabilities for the cost-effectiveness analysis are presented in Table 1. More details about the sources of key model inputs, along with justifications, can be found in Supplemental Material Table S1. In each study strategy, the probabilities of switching, titration, waiting, discontinuation, and suicide attempts were assumed to be identical for both cytochromes, regardless of the metabolizer class. The probabilities of dosage titration, switching or waiting due to side effects or no treatment effect, and suicide attempts were derived from the cost-effectiveness study by Carta et al. (2022). Short-term model probabilities are reported in the Supplemental Material Table S2.

**Table 1.** Key model input parameters and weighted probabilities.

Input parameter				Base-case	Uncertainty range	Uncertainty sampling distribution	Source
**Prevalence of phenotype mutations**	CYP2D6		PM	1.89%	Fixed	–	Jithesh et al. (2022)
			IM	23.28%	Fixed	–	
			URM	8.55%	Fixed	–	
	CYP2C19		PM	1.87%	Fixed	–	
			IM	19.80%	Fixed	–	
			URM	6.53%	Fixed	–	
			RM	29.84%	Fixed	–	
	Proportion of CYP2D6 and CYP2C19 mutation detected in the intervention cohort			100%	Fixed	–	Assumption
	Specificity of screening			100%	Fixed	–	Assumption
	Sensitivity of screening			100%	Fixed	–	Assumption
**Short-term model**							
**PGx (CYP2D6)**	Probability (allele carrier)	PM	Response	0.370	95% CI	Beta	Rush et al. (2006) (STAR*D trial)
			Relapse	0.099			Sim et al. (2015)
		IM	Response	0.370			Rush et al. (2006) (STAR*D trial)
			Relapse	0.099			Sim et al. (2015)
		URM	Response	0.350			Sluiter et al. (2019)
			Relapse	0.099			Sim et al. (2015)
	Probability (allele non-carrier)	PM	Response	0.370			Rush et al. (2006) (STAR*D trial)
			Relapse	0.099			Sim et al. (2015)
		IM	Response	0.370			Rush et al. (2006) (STAR*D trial)
			Relapse	0.099			Sim et al. (2015)
		URM	Response	0.350			Sluiter et al. (2019)
			Relapse	0.099			Sim et al. (2015)
**SoC**	Probability (allele carrier)	PM	Response	0.370			Rush et al. (2006) (STAR*D trial)
			Relapse	0.233			Sim et al. (2015)
		IM	Response	0.370			Rush et al. (2006) (STAR*D trial)
			Relapse	0.233			Sim et al. (2015)
		URM	Response	0.350			Sluiter et al. (2019)
			Relapse	0.233			Sim et al. (2015)
	Probability (allele non-carrier)	PM	Response	0.370			Rush et al. (2006) (STAR*D trial)
			Relapse	0.233			Sim et al. (2015)
		IM	Response	0.370			Rush et al. (2006) (STAR*D trial)
			Relapse	0.233			Sim et al. (2015)
		URM	Response	0.350			Sluiter et al. (2019)
			Relapse	0.233			Sim et al. (2015)
**PGx (CYP2D6)**	Probability (allele carrier and non-carrier)	PM	Side effect	0.490			Shams et al. (2006)
		IM		0.490			Shams et al. (2006)
		URM		0.300			Shams et al. (2006)
**SoC (CYP2D6)**	Probability (allele carrier and non-carrier)	PM		0.900			Shams et al. (2006)
		IM		0.700			Shams et al. (2006)
		URM		0.300			Shams et al. (2006)
**PGx (CYP2C19)**	Probability (allele and non-carrier)	PM	Response	0.580			Carta et al. (2022)
			Relapse	0.099			Sim et al. (2015)
		IM	Response	0.480			Carta et al. (2022)
			Relapse	0.099			Sim et al. (2015)
		URM	Response	0.370			Fabbri et al. (2021)
			Relapse	0.099			Sim et al. (2015)
		RM	Response	0.370			Rush et al. (2006) (STAR*D trial)
			Relapse	0.099			Sim et al. (2015)
**SoC**	Probability (allele and non-carrier)	PM	Response	0.480			Fabbri et al. (2021)
			Relapse	0.230			Sim et al. (2015)
		IM	Response	0.370			Fabbri et al. (2021)
			Relapse	0.230			Sim et al. (2015)
		URM	Response	0.370			Fabbri et al. (2021)
			Relapse	0.230			Sim et al. (2015)
		RM	Response	0.370			Fabbri et al. (2021)
			Relapse	0.230			Sim et al. (2015)
**PGx (CYP2C19)**	Probability (allele carrier and non-carrier)	PM, IM, RM	Side effect	0.490			Carta et al. (2022)
		URM		0.490			Fabbri et al. (2021)
**SoC (CYP2C19)**	Probability (allele carrier)	PM		0.550			Fabbri et al. (2021)
		IM		0.510			Fabbri et al. (2021)
		URM		0.490			Fabbri et al. (2021)
		RM		0.900			Fabbri et al. (2021)
**SoC (CYP2C19)**	Probability (allele non- carrier)	PM		0.550			Fabbri et al. (2021)
		IM		0.510			Fabbri et al. (2021)
		URM		0.490			Fabbri et al. (2021)
		RM		0.490			Fabbri et al. (2021)
**PGx (CYP 2D6 and 2C19)**	All metabolizers, carrier, and non-carrier		Switching to alternative	0.680			Bradley et al. (2018)
			Titrating dose	0.200			Bradley et al. (2018)
			Observation	0.250			
			Discontinuation	0.260			
			Suicide attempt	0.060			
			Suicide death	0.006			
**SoC (CYP 2D6 and 2C19)**	All metabolizers, carrier, and non-carrier		Switching to alternative	0.450			Bradley et al. (2018)
			Titrating dose	0.240			
			Observation	0.250			
			Discontinuation	0.080			
			Suicide attempt	0.060			
			Suicide death	0.006			
**CYP2D6**	**Costs**				95% CI (multivariate analysis)	Gamma with 95% CI	HMC
**PM**			Acquisition cost SoC first-line and alternative	25	±25% for cost of panel genotyping test (univariate analysis)	Triangular with ±25%	
**IM**			Acquisition cost SoC first-line and alternative	25			
**URM**			Acquisition cost SoC first-line and alternative	25			
**PM**			Acquisition cost PGx first-line and alternative	12			
**IM**			Acquisition cost PGx first-line and alternative	12			
**URM**			Acquisition cost PGx first-line and alternative	24			
**PM**			Acquisition cost SoC discontinuation	6			
**IM**			Acquisition cost SoC discontinuation	6			
**URM**			Acquisition cost SoC discontinuation	6			
**PM**			Acquisition cost PGx discontinuation	6			
**IM**			Acquisition cost PGx discontinuation	6			
**URM**			Acquisition cost PGx discontinuation	6			
**PM**			Acquisition cost SoC increased dose	47			
**IM**			Acquisition cost SoC increased dose	47			
**URM**			Acquisition cost SoC increased dose	47			
**PM**			Acquisition cost PGx increased dose	25			
**IM**			Acquisition cost PGx increased dose	25			
**URM**			Acquisition cost PGx increased dose	47			
**CYP2C19**							
**PM**			Acquisition cost SoC first-line and alternative	76			
**IM**			Acquisition cost SoC first-line and alternative	76			
**RM**			Acquisition cost SoC first-line and alternative	76			
**URM**			Acquisition cost SoC first-line and alternative	76			
**PM**			Acquisition cost PGx first-line and alternative	38			
**IM**			Acquisition cost PGx first-line and alternative	38			
**RM**			Acquisition cost PGx first-line and alternative	76			
**URM**			Acquisition cost PGx first-line and alternative	76			
**PM**			Acquisition cost SoC discontinuation	38			
**IM**			Acquisition cost SoC discontinuation	38			
**RM**			Acquisition cost SoC discontinuation	38			
**URM**			Acquisition cost SoC discontinuation	38			
**PM**			Acquisition cost PGx discontinuation	19			
**IM**			Acquisition cost PGx discontinuation	19			
**RM**			Acquisition cost PGx discontinuation	38			
**URM**			Acquisition cost PGx discontinuation	38			
**PM**			Acquisition cost SoC increased dose	152			
**IM**			Acquisition cost SoC increased dose	152			
**RM**			Acquisition cost SoC increased dose	152			
**URM**			Acquisition cost SoC increased dose	152			
**PM**			Acquisition cost PGx increased dose	108			
**IM**			Acquisition cost PGx increased dose	108			
**RM**			Acquisition cost PGx increased dose	152			
**URM**			Acquisition cost PGx increased dose	152			
**Both groups**			Lab and screening	560			
**Side effects management, both groups**			Sexual dysfunction	208			
			Hyponatremia	129			
			Arrythmia	389			
			Serotonin syndrome	103			
			Bruxism	–			
			Insomnia	12			
**Events, both groups**			Relapse*	954			
			Suicide death*	15			
			Suicide attempt*	390			
			Non-suicide death	1020			
			Survival	1300			
**Follow-up, both groups**			Consultation visit	1000			
**Genotyping test, PGx**			Panel genotyping test	540			
**Long-term model**							
**PGx**	Transition probabilities		Response with or without side effects and without relapse	0.1180	95% CI	Beta	Decision-tree
			Relapse with and without side effects	0.0949			
			Relapse with and without side effects	0.0229			
			No response	0.0965			
			Suicide death with and without side effects and without response	0.1175			
			Death from non-suicide causes	0.1180			
**SoC**			Response with or without side effects and without relapse	0.1170			
			Relapse with and without side effects	0.2062			
			Relapse with and without side effects	0.0572			
			No response	0.0965			
			Suicide death with and without side effects and without response	0.1176			
			Death from non-suicide causes	0.1170			
**Both groups**	Utility of response		40–49 years	0.871			Sullivan and Ghushchyan (2006)
			50–59 years	0.842			
			60–69 years	0.823			
			70–79 years	0.79			
			≥80 years	0.736			
	Utility of relapse		Not age specific	0.55			Carta et al. (2022)
	Utility of no response		Not age specific	0.48			
**PGx**	Costs		Acquisition cost	648	95% CI	Gamma	HMC
**SoC**			Acquisition cost	595			
**Both groups**			Lab and screening	560			
**Both groups**			Relapse event*	1908			
**Both groups**			Suicide death event*	29			
**Both groups**			Suicide attempt event*	779			
**Both groups**			Non-suicide death event	2040			
**Both groups**			Survival event	2600			
**Both groups**			Follow up visit	500			
**Both groups**	**Discount rate**		Beyond 1 year	3%	±25%	Triangular	Haacker et al. (2020)

Note: PGx: pharmacogenetics testing; PM: poor metabolizer; IM: intermediate metabolizer; RM: rapid metabolizer; URM: ultra-rapid metabolizer; SoC: standard of care; CI: confidence interval. HMC: Hamad Medical Corporation. #Costs are in Qatari Riyal. 3.64 QAR = 1 USD. *Adapted based on the literature.

#### Long-term Markov model

The probabilities for all outcomes were obtained from the results of our decision-tree analysis. In this model, 3-month transition probabilities were derived from event rates. For the first cycle, the risk of events was based on event probabilities estimated from the decision tree and local reports (Births & Deaths In the State of Qatar, 2016; Qatar. Institute for Health Metrics and Evaluation, 2021). For all subsequent cycles, transition probabilities were adjusted based on age-related trends estimated by the Ministry of Development Planning and Statistics, Qatar (Births & Deaths In the State of Qatar, 2019; Qatar. Institute for Health Metrics and Evaluation, 2021), to account for the risk of death due to mental and non-mental health causes according to age-specific mortality rates. To estimate mortality rates by single year of age, mortality rates for an age group were plotted against the midpoint age for the age group (e.g. 52 for the age group 50–54 years), and polynomial functions were applied to model the mortality rates for age in single years. Key input data are shown in Table 1, and additional information can be found in the Supplemental Material Table S1 and S3.

### Study outcomes

The definition of therapy success was adapted to local interests by decision-makers at the Mental Health Hospital in Qatar.

#### Short-term decision-analytic model

The primary outcome, referred to as success, was measured by the incremental cost-effectiveness ratio (ICER), which was defined as the cost per response without relapse, without side effects at 6 weeks. A secondary outcome was also sought, which was the ICER defined as the cost per response without relapse, with or without side effects at 6 weeks.

#### Long-term Markov model

The primary outcomes were the ICER, defined as the cost per quality-adjusted life year (QALY) gained and also as the cost per year of life saved.

### Cost calculation

As per the study perspective, the costs of the direct medical resources were considered in the model, which encompassed expenses related to genetic screening, acquisition of antidepressants, alternative therapy, management of side effects, events, screening, radiology and laboratory tests, and consultation visits. It is important to note that only costs associated with antidepressant therapy were considered. Indirect costs and potential productivity losses were not included due to a lack of available data.

Resource utilisation was estimated based on the Canadian Network for Mood and Anxiety Treatments (CANMAT) guideline (Kennedy et al., 2016), which is the guideline that the HMC practice is primarily based on. Then, a panel of experts, consisting of clinical pharmacists experienced in mental health and its treatments, was consulted to verify the pattern of resource use.

The price for PGx testing utilised using multi-gene panel testing was sourced from the Qatar Genome Program while medication prices were obtained from the Pharmacy Department of HMC. The cost of initial therapies included the complete cost of first-line treatments for the entire duration of the regimen, based on the prescribing pattern in Qatar (Elbakary et al., 2021). For patients who discontinued treatment, the cost calculation included the cost of therapy until discontinuation (assuming half of a treatment cycle, 3 weeks), in addition to the cost of alternative therapy (6 weeks). The model assumed that the distribution of alternative therapies was the same as that for first-line therapies.

The costs associated with events, side effects management, screening, radiology, laboratory tests, and consultation visits were obtained from HMC Costing and Finance Department. The cost of managing side effects was determined based on the resources consumed in side effects management, including medications, laboratory orders, and hospital visits. The cost of consultation (follow-up) visits, screening, radiology, and laboratory tests was determined based on the expenses associated with the tests ordered throughout the follow-up period.

The costs of suicide death and relapse events were not available specifically for Qatar. Therefore, the cost of these events was estimated based on data from the literature, from cost data in the United States (US) and Belgium (Demyttenaere et al., 2005; Touya et al., 2022), which underwent cost adaptation, *Vide Infra*.

For cost-effectiveness analysis in Qatar, consistent with the literature and the WHO recommendations, a willingness-to-pay (WTP) threshold of USD 150,000 per additional year of life saved and per QALY gained was used as a reference threshold (Abushanab et al., 2021; Al-Badriyeh et al., 2022; Kaddoura et al., 2022). All outcomes were discounted at an annual rate of 3%, following the recommendation of the Second Panel on Cost-Effectiveness in Health and Medicine (Haacker et al., 2020). Discounting is applied in health economic models with long-term horizons to reflect the lost value of future outcomes (Attema et al., 2018). Cost calculation was expressed in Qatari Riyal (QAR) and based on the financial year 2024/25.

#### Cost adaptation

To convert costs from Belgian Euro or United States dollar (USD) to QAR, a cost-adjustment method was employed (Ademi et al., 2018). This method considers not only the currency value but also corrections for differences in health resource utilisation and the prices of healthcare services, as well as adjustments for inflation. Data on healthcare expenses per capita and the prices of healthcare services were obtained from the Organization for Economic Cooperation and Development (OECD). Further details about the cost-adaptation process can be found in the Supplemental Material Table S5.

According to the decision analysis principles of modelling (Briggs et al., 2006), the overall cost of a study approach, encompassing all health states and their associated uncertainties, is determined by summing the ‘proportional costs’ of the different health states. The proportional cost of a health state is calculated by multiplying the ‘cost of the health state’ by the ‘probability of the health state’.

Supplemental Material Table S4 provides detailed data obtained from the CANMAT guidelines and the study expert panel.

### Utilities

The effectiveness of the intervention in the Markov model was measured using QALYs as the outcome measure. A QALY is calculated by multiplying the quality of life (utility score) when in a health state by the life years spent in the health state. A utility score of one indicates perfect health, while zero represents death (Whitehead & Ali, 2010). Utilities for response were extracted from Sullivan and Ghushchyan (2006) and were age-specific, while utilities for relapse and no response were obtained from Kuyken et al. (2008). The utility value for a suicide attempt was assumed to be zero. All utilities were measured using the EuroQol five-dimension questionnaire (Table 1). Further details about the sources of these studies can be found in the Supplemental Material Table S1.

### Sensitivity analyses

The sensitivity analyses included a univariate sensitivity analysis, a multivariate sensitivity analysis, and scenario analyses.

The univariate analysis entailed re-running the model several times, where the impact of changes in specific input variables on the output results was examined, with one input variable being changed at a time. In this analysis, the cost of PGx-guided testing and the discount rate were varied by ±25% using triangular distribution. The multivariate sensitivity analysis accounted for re-running the model several times, where the uncertainty of multiple parameters in the model was examined by simultaneously changing all input variables. For each model iteration, uncertain parameters were assigned a random value from a pre-defined uncertainty range based on a sampling distribution. Clinical, cost, and utility parameters were varied using the 95% confidence intervals (CI) for the parameters. Probability rates and utilities were sampled using beta distributions, while costs were sampled using gamma distributions (Briggs et al., 2006). The results of these iterations were presented in a cost-effectiveness acceptability curve (CEAC). The range of each parameter and the distributions applied are presented in Table 1. A Monte Carlo simulation of 50,000 iterations was performed using @Risk-5.7® (Palisade Corporation, NY, US) to assess the uncertainty surrounding the point estimates.

Additionally, several scenario analyses were performed. These included shortening the time horizon to 5 years, removing age-related trends from the transition probabilities in the Markov model, removing the discount rate in the Markov model, changing the alternative therapy to SNRIs, such as duloxetine, and applying half-cycle correction.

### Model validation

To ensure the accuracy and reliability of the model, various validation measures were undertaken. These included assessing the consistency of assumptions and inputs with current literature, and testing the robustness of the results against variations in key parameters. The Assessment of the Validation Status of Health-Economic Decision Models Checklist (Vemer et al., 2016) was used to evaluate the validity of the model. Additionally, a team consisting of practitioners and health economists reviewed the assumptions, model structure, and findings.

To further enhance the face validity of the modelling approach and data sources, independent checks were conducted by DA-B and DA in the Excel sheet to identify any potential modelling errors. The guidance provided by the harmonisation and improvement of economic evaluations of personalised medicine (Vellekoop et al., 2021) and the National Institute for Health and Care Excellence (NICE) technology appraisal guidance for the UK (National Institute for Health and Care Excellence, 2022) were taken into consideration. Moreover, all methods and analyses adhered to the recommendations outlined by the Second Panel on Cost-Effectiveness in Health and Medicine and the Consolidated Health Economic Evaluation Reporting Standards (CHEERS) statement (Husereau et al., 2022) (Supplemental Material Table S6).

### Ethical approval and consent procedures

As this study is a literature-based simulation study and does not involve direct interaction or data collection from patients. Ethics approval and informed consent procedures were not applicable.

## Results

### Base-case analysis

#### Short-term decision-analytic model

The mean difference in response, without side effects and without relapse after six weeks, between PGx-guided testing and SoC was 0.10 (95% CI, 0.09–0.15), favouring PGx-guided testing. Similarly, the mean difference in response, with or without side effects and without relapse, between PGx-guided testing and SoC after six weeks was 0.05 (95% CI, 0.04–0.06), also favouring PGx-guided testing. The PGx-guided testing was associated with lower total costs (QAR 25,503 versus QAR 27,792) compared to the SoC. Therefore, PGx-guided testing was dominant over the SoC, resulting in a mean cost-saving of QAR 2,289 (95% CI, −22,654–26,340). The base-case analysis results are presented in Table 2.

**Table 2.** Results from the base-case analysis.

Outcome	PGx	Standard of care	Difference in favour of PGx (95% CI)
**Short-term model**			
Response without side effects and without relapse	0.76	0.657	0.10333 (0.09–0.15)
Response with or without side effects and without relapse	0.53	0.47	0.05342 (0.04–0.06)
Total cost	25,503	27,792	2,289 (−22,654–26,340)
ICER (Response without side effects and without relapse)	PGx dominance^a^		
ICER (Response with or without side effects and without relapse)	PGx dominance^a^		
**Long-term model**			
Response with or without side effects and without relapse	28	22	7
Relapse (with and without side effects)	6	26	20
No response	87	95	8
Suicide death with and without side effects and without response	233	237	4
Death from non-suicide causes	17,595	17,873	278
Years of life saved	1335	1207	127
QALY	927	866	61
Cost of events	58,980	97,208	38,228
Cost of medications	16,772	23,699	6,927
Cost screening and lab	21,747	22,307	560
Cost of follow up	19,423	19,923	500
Total cost	116,922	163,137	46,215
ICER/ Years of life saved	PGx dominance^a^		
ICER/QALY	PGx dominance^a^		

Note: PGx: pharmacogenetics testing; SoC: standard of care; CI: confidence interval; QALY: quality adjusted life year; ICER: incremental cost effectiveness ratio. #Costs are in Qatari Riyal. 3.64 QAR = 1 USD.

^a^ Cost-saving or ‘Dominant’ strategies produce negative ICERs, highlighting the fact that produce better health and economic outcomes. The values of negative ICERs are not reported in cost-effective analysis. All costs are presented in 2024 Qatari Riyals. Totals may not add up to the unit due to rounding.

#### Long-term Markov model

Compared to the current SoC, PGx-guided testing would lead to the additional saving of 127 years of life (95% CI −367–707) (0.1/person) and a gain of 61 QALYS (95% CI −285–457) (0.06/person). Additionally, PGx-guided testing was associated with lower total direct healthcare costs, with QAR 116,922 compared to QAR 163,137 with the current SoC. This translates to a cost saving of QAR 46,215 (95% CI −15,744–101,758) (Table 2).

## Sensitivity analyses

### Univariate sensitivity analysis

#### Short-term decision-analytic model

Varying the cost of genotyping test had a negligible impact on the cost saving, ranging from QAR 2,150 to QAR 2,450.

#### Long-term Markov model

Varying the discount rate did not influence the overall cost saving, ranging from QAR 45,300 to QAR 45,750.

### Multivariate sensitivity analysis

#### Short-term decision-analytic model

**Figure 2.**
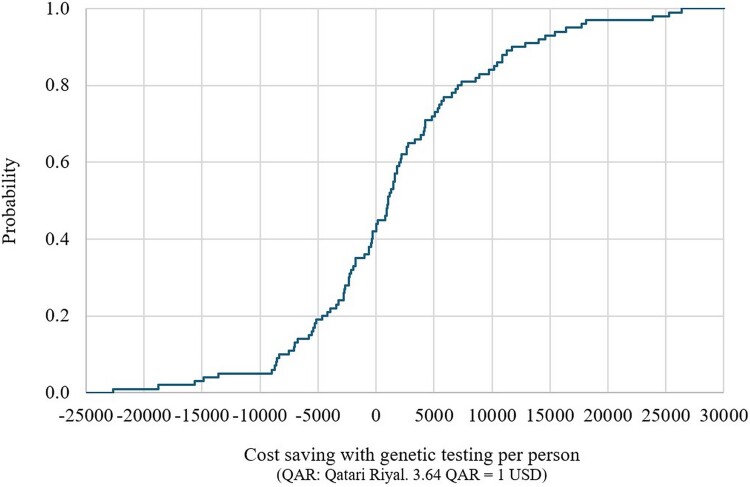
Short-term cost-saving probability curve with genetic testing, for the response without side effects and without relapse outcome (multivariate sensitivity analysis).

**Figure 3.**
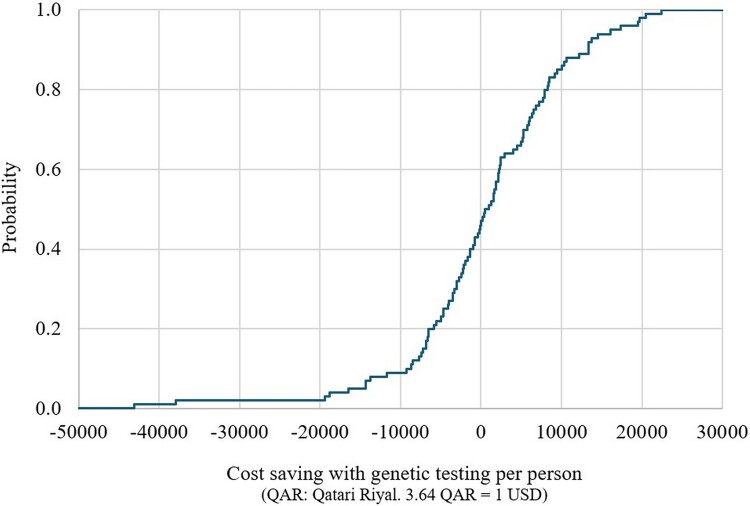
Short-term cost-saving probability curve with genetic testing, for the response with or without side effects and without relapse outcome (multivariate sensitivity analysis).

**Figure 4.**
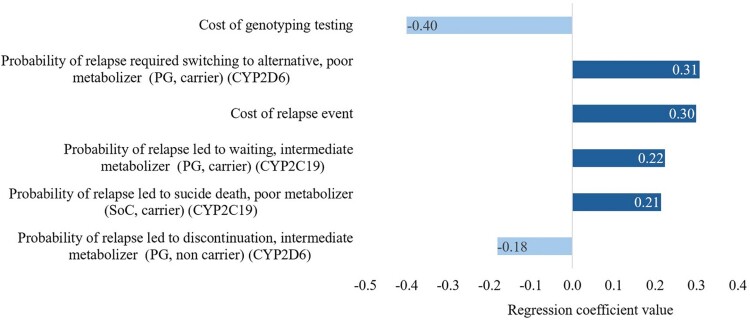
A regression tornado diagram indicating the main drivers of model variables on short-term cost saving, the response without side effects and without relapse outcome.

**Figure 5.**
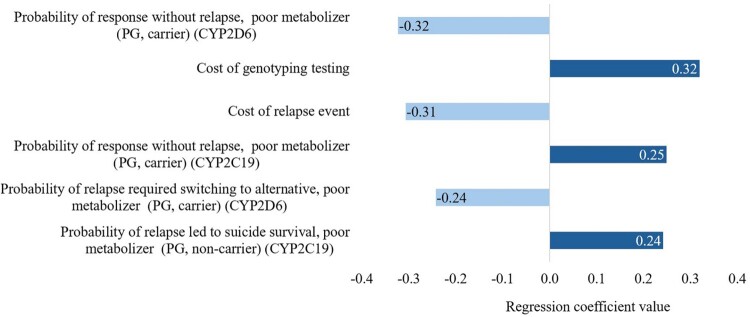
A regression tornado diagram indicating the main drivers of model variables on short-term cost saving, the response with or without side effects and without relapse outcome.

The CEPC analysis showed that the PGx-guided testing resulted in cost savings (dominance) in approximately 40% and 43% and cost-effectiveness in around 42% and 39% of the simulated cases for the response without side effects and without relapse outcome, and the response with or without side effects and without relapse outcome, respectively (Figures 2 and 3). The regression tornado analysis identified the cost of genotyping testing, the probability of relapse requiring switching to an alternative, poor metabolizer (PGx-guided testing, carrier, CYP2D6), and the probability of response without relapse, poor metabolizer (PGx-guided, carrier, CYP2D6) as key drivers influencing the cost-saving outcomes (Figures 4 and 5).

#### Long-term Markov model

**Figure 6.**
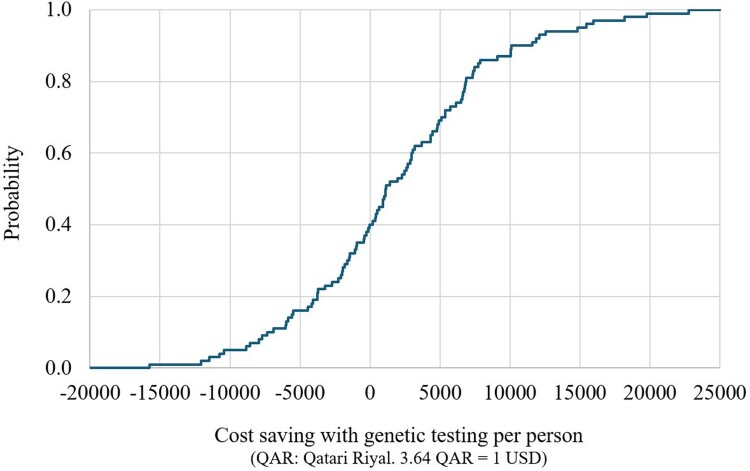
Cost-saving probability curves with genetic testing (multivariate sensitivity analysis).

**Figure 7.**
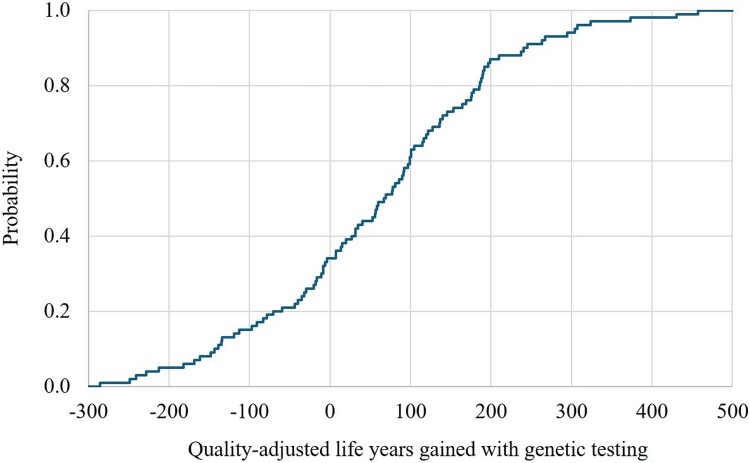
Quality-adjusted life years gain probability curve with genetic testing (multivariate sensitivity analysis).

**Figure 8.**
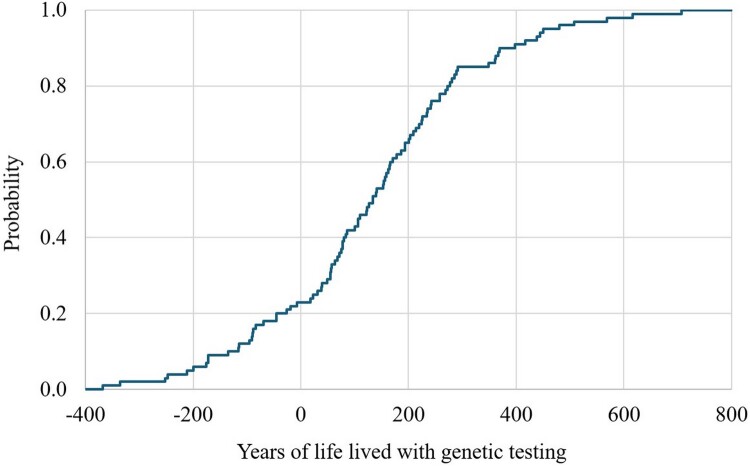
Years of life saved probability curve with genetic screening (multivariate sensitivity analysis).

**Figure 9.**
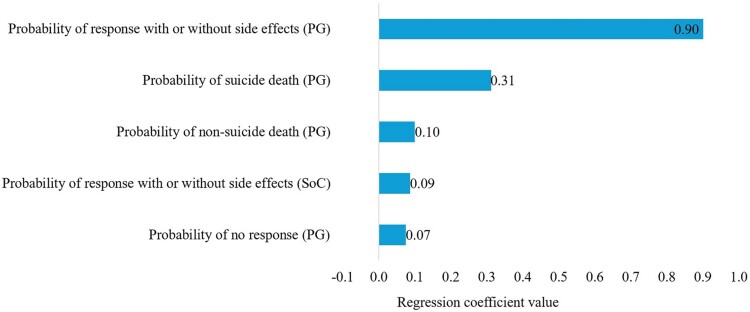
A regression tornado diagram indicating the main drivers of model variables on long-term cost saving.

The CEPC analysis showed that the PGx-guided testing was cost saving (dominance) in 40% of the simulated cases and was cost-effective in 53% of the cases (Figure 6). For years of life saved and QALYs gained, the CEPC analysis demonstrated that PGx-guided testing was cost-saving in 21% and 35% of the simulated cases, respectively (Figures 7 and 8). The regression tornado analysis revealed that the probability of response with or without side effects and the probability of suicide death (PGx-guided testing) were the key drivers influencing the cost-saving outcome (Figure 9).

### Scenario analysis

The overall base-case cost saving was maintaied across the different scenarios. The results of all scenarios are pesented in Table 3.

**Table 3.** Results from the scenario analysis.

Scenario	Short-term model	Long-term model
	Cost saving, QAR	Cost saving, QAR
Base-case analysis	2289	46,215
Considering undiscounted outcomes 0%	2289	46,410
Removing age-related trends from the transition probabilities in Markov model	2289	43,267
Shortening time horizon to 5 years	2289	40,139
Changing alternative to duloxetine	2251	46,215
Applying half cycle correction	2289	46,215

Note: QAR: Qatari Riyal. 3.64 QAR = 1 USD.

### Model validation

Validation checklists are provided in the Supplemental Material Table S7.

## Discussion

Many patients with moderate-to-severe MDD initially do not respond to standard treatments, leading to a trial-and-error approach where multiple medications are sequentially tried. This process often results in significant delays in achieving a response, causing prolonged suffering, increased healthcare costs, and higher risk of suicide. Here, there is growing evidence supporting the use of PGx-guided treatment to expedite the identification of an effective treatment regimen (Bradley et al., 2018; Hall-Flavin et al., 2012; Winner et al., 2013).

Our cost-effectiveness study aimed to assess the economic implications of PGx-guided testing, particularly targeting CYP2D6 and CYP2C19 genes, in MDD patients initiating antidepressant therapy. In our analysis, PGx-guided strategy demonstrated superior outcomes compared to SoC. In the short-term model, it showed a mean response rate improvement of 0.10 (95% CI: 0.09–0.15) without side effects, and 0.05 (95% CI: 0.04–0.06) when considering side effects. Additionally, PGx-guided strategy resulted in lower total costs (QAR 25,503 versus QAR 27,792), leading to a mean cost saving of QAR 2,289 (95% CI: −22,654 to 26,340). In the long-term Markov model, PGx-guided strategy led to an estimated 127 years of life saved and 61 QALYs gained, with total costs of QAR 116,922 compared to QAR 163,137 for SoC, resulting in a cost saving of QAR 46,215 (95% CI: −15,744 to 101,758). Overall, these findings highlight the effectiveness and cost-effectiveness of PGx-guided strategy over SoC, supporting its consideration in the Qatari clinical practice. Among the parameters considered, in addition to the genotyping cost, the cost of relapse events and the probability of relapse requiring a switch to an alternative treatment regimen were found to be the most influential factors affecting the cost savings. The scenario analysis revealed that even when shortening the time horizon to 5 years, the cost-saving benefits of PGx-guided were maintained.

In the short-term analysis, we observed that the parameters relating to PM and IM had the most influence on the cost savings; whereby, unlike other metabolizer classes, the PM and IM individuals in the population received dose adjustments based on the genotyping results, which likely contributed to the observed cost savings.

The wide range of the CIs for cost savings in our study reflects the variability in several key parameters, including the costs associated with relapse events and the likelihood of patients needing to switch to alternative treatment regimens. This variability highlights the uncertainty inherent in modelling simulation-based clinical outcomes and emphasises the importance of interpreting these results with caution. For example, in the short-term decision-analytic model, the mean difference in response without side effects and relapse after six weeks favoured PGx-guided strategy, with a mean difference of 0.10 (95% CI, 0.09–0.15). Similarly, the mean difference in response with or without side effects was 0.05 (95% CI, 0.04–0.06), also favouring the PGx-guided approach. Despite the positive responses, the mean cost-saving associated with PGx-guided strategy was QAR 2,289 (95% CI, −22,654–26,340), showing that while cost savings are anticipated, there remains a potential for increased costs in certain scenarios, as reflected by the negative lower bound of the CI. This aspect emphasises the inherent uncertainty in the estimates. In the long-term Markov model, PGx-guided strategy was associated with significant potential benefits, including an additional saving of 127 years of life (95% CI, −367–707) and a gain of 61 QALYs (95% CI, −285–457). The direct healthcare costs for PGx-guided strategy were lower at QAR 116,922 compared to QAR 163,137 with the SoC, translating to a cost saving of QAR 46,215 (95% CI, −15,744–101,758). The broad range of CIs in both models reflects the uncertainty in these estimates, particularly regarding the longevity and quality of life improvements associated with PGx-guided strategy.

There are several potential reasons for the projected cost savings observed in this study. Firstly, the evaluated panel test incorporates a multi-variant approach, which may be more cost-effective compared to conducting a series of single tests. Secondly, the integrated report from the panel test offers comprehensive findings from multiple gene variants, facilitating the stratification of medications based on various outcomes, such as response with or without side effects, response with or without relapse, and no response, thereby aiding informed decision-making. However, considering the complexity of interpreting gene-panel tests, striking a balance between reporting and interpreting genetic analysis remains a concern for medical geneticists to ensure practical interpretability for busy clinicians and patients (Green et al., 2013).

Despite the existence of studies conducted worldwide, the generalizability of these is limited by significant variations in the frequency of different metabolising phenotypes among populations, as well as the heterogeneity of health systems. Nevertheless, our findings are consistent with previous cost-effectiveness studies focusing on PGx-guided psychiatric medications, which also demonstrated favourable results, predicting cost savings (Groessl et al., 2018; Hornberger et al., 2015; Najafzadeh et al., 2017; Tanner et al., 2020). However, it is worth noting that much of the previous studies in this area were funded by PGx test manufacturers and were not conducted within the context of Qatar.

Our results are consistent with the findings of Ghanbarian et al. (2023), who estimated substantial savings of CA$ 956 million (approximately QAR 2.5 billion) for the Canadian healthcare system through the use of PGx-guided strategy, equating to CA$ 4,926 (around QAR 13,226) saved per patient. Their study highlighted that these savings were largely attributed to preventing transitions to refractory depression. While their reported savings per patient are considerably higher than ours, the differences may stem from higher frequencies of CYP2D6 and CYP2C19 metabolizer phenotypes in the Canadian population, which may enhance the effectiveness of PGx-guided strategy relative to our Qatari population.

While both our study and the study by Ghanbarian et al. (2023) incorporated panel testing, considered drug-specific characteristics and a 20-year time horizon, there were several differences in the methodology. Our analysis included a decision tree analysis to evaluate the short-term impact of PGx-guided testing, which demonstrated the dominance of the testing strategy over SoC. Additionally, the Ghanbarian et al. simulation was based on weekly follow-up cycles, whereas our Markov model operated on 3 monthly cycles. While a shorter cycle allows for more frequent updates and transitions within the model, it may not fully capture the complexities of depression, which is a chronic condition that evolves over longer durations. Relative to a 1-week cycle, a longer cycle, such as 3 monthly, provides a more comprehensive representation of disease progression, allowing for a better understanding of symptom variations, treatment outcomes, and relapse rates among different patient subgroups.

Groessl et al. (2018) conducted another cost-effectiveness analysis, from a societal perspective, focusing on treatment-naive patients or patients with inadequately controlled MDD. Their findings showed that over a 3-year period, the PGx treatment group had an estimated cumulative effect of 2.07 QALYs, while the SoC group had 1.97 QALYs. In contrast, our long-term Markov model suggested a higher gain of 61 QALYs associated with PGx-guided strategy. This substantial difference in QALYs may reflect the broader scope of our analysis, which included multiple health states and a longer time horizon. Additionally, Groessl et al. (2018) reported that the PGx-guided group resulted in cost savings of US$ 2,918 (QAR 10,627) in direct medical costs. This figure is lower than those reported in our analysis, likely due to their more limited range of health outcomes and shorter 3-year analysis period.

While the results are consistent with ours, favouring PGx testing, the studies are not comparable. Unlike our study, which employed a decision tree analysis followed by Markov modelling, Grossel et al. focused on three health states only: response, remission, and survival. Our model, on the other hand, incorporated additional states such as response with or without side effects, relapse with or without side effects, and suicide death. Importantly, the combination of decision trees and Markov models in our study provides a comprehensive framework for analysing the full spectrum of decision-making in depression. This integrated approach allows for a holistic assessment of treatment strategies, resource allocation, and cost-effectiveness. Furthermore, Grossel et al. used a time horizon of 3 years only and their analysis was class based, different from our and the Ghanbarian et al. (2023) studies, which were drug specific.

Another study, conducted by Hornberger et al. (2015), also examined the cost-effectiveness of PGx in the management of treatment-resistant depression from a societal perspective, similar to the study by Groessl et al. (2018). Hornberger et al. reported that the PGx-guided testing improved the treatment response rate by 70% compared to SoC, resulting in a 0.316 QALYs increase, which is lower than our findings. With this, the PGx-guided testing was projected to save US$ 3,711 (QAR 13,515) in patient direct medical costs, which is also lower than the cost savings observed in our study (QAR 46,215), and US$ 2,553 (QAR 9,297) in patient work productivity costs over lifetime. The differences in results can be attributed to several factors. Our analysis incorporated data from a variety of studies to capture the full range of consequences associated with gene mutations, while Hornberger et al. relied on response rates from a meta-analysis they conducted, which included three smaller studies with sample sizes of 49, 44, and 165. Additionally, our model analysed data reflecting a broader range of depression, including treatment-naive patients, whereas the prior study focused only on treatment-resistant depression. It is unclear how studying treatment-naive versus treatment-resistant patients affects the cost-effectiveness results, but evidence suggests that treatment-resistant patients have diminishing chances of achieving remission with each failed treatment attempt (Altar et al., 2013). This implies that the cost savings and effectiveness of PGx testing may be slightly greater in the treatment-naive group. Another difference is that our study conducted a scenario analysis with a 5-year time horizon, focusing on differences between groups during the catch-up period. This period refers to a phase where the SoC might achieve response rates comparable to those guided by PGx, as suggested in the medical literature (Rush et al., 2006). In contrast, the study by Hornberger et al. addressed cumulative cost-effectiveness over the patient's lifetime, which served as the base-case scenario in our model.

From an Italian perspective, Serretti et al. (2011), reported that the use of PGx testing was associated with increases in antidepressant response (0.062 quality-adjusted life weeks (QALWs)) and tolerability (0.016 QALWs), with an ICER of US$ 2,890 (QAR 10,525). However, it is important to note that this analysis was limited to a duration of 12 weeks, which may significantly underestimate the actual QALYs and cost saving benefits of PGx testing. This limitation introduces uncertainty regarding the true trajectory of a patient's response over the first year and beyond. Furthermore, the Serretti et al. study focused exclusively on patients who received citalopram or bupropion, which are different medications compared to the broader range of medications considered in our study, affecting cumulative treatment outcomes. Another important distinction is that our model takes a comprehensive approach by considering various health states such as side effects, relapse, lack of response, discontinuation, and switching to alternative treatments. In contrast, the Serretti et al. study was limited to remission and side effects.

The wide CIs reported in our findings, particularly for cost savings (QAR 2,289 with a 95% CI of −22,654 to 26,340 for the short-term and QAR 46,215 with a 95% CI of −15,744 to 101,758 for the long-term), reflect the inherent variability in modelling health outcomes, especially in a complex condition like MDD. Such variability is not uncommon in health economic evaluations (Briggs & Gray, 1999; Jackson et al., 2009), particularly those including PGx testing, which can be further affected by multiple factors, including genetic variability, treatment choices, and patient adherence. For instance, the reliance on certain assumptions, such as the 100% diagnostic rate for PGx-guided strategy and the fixed probabilities of treatment responses, could contribute to this uncertainty. However, the extent of the CIs in our study does raise some concerns regarding the reliability of the results. The possibility of negative cost savings indicates scenarios where the PGx-guided strategy might not be beneficial, highlighting the need for a cautious interpretation of our findings. Furthermore, the differences in methodologies, such as the choice of modelling approaches and the time horizon used, can significantly influence the level of uncertainty. For example, while our use of a Markov model allows for a more comprehensive representation of disease progression, it also adds complexity that may introduce additional variability. To enhance the reliability of our results, future research should focus on refining the input parameters and utilising real-world data to validate assumptions.

While our findings may need to be replicated in a real-world setting, our study provides evidence that can help overcome one of the main current barriers to the implementation of PGx-guided in Qatar, which is the cost of the strategy. We also believe that our findings have practical implications for clinical practice, particularly for patients aged over 60 years, who often receive polypharmacotherapy, and where multiple drug-gene interactions can enhance how beneficial and cost-effective the PGx-guided is (Altar et al., 2015; Biskupiak et al., 2015). Additionally, despite that our study reveals that PGx-guided strategy not only has the potential to enhance treatment outcomes but also reduce overall healthcare costs on the long term, the level of uncertainty observed, particularly in the CIs surrounding cost savings highlights the need for further validation of these findings in diverse clinical settings. Investing in pilot programmes would allow for real-world implementation of PGx-guided strategy, allowing researchers to gather data on its effectiveness and cost implications across varied patient populations. Such studies could help refine the key variables, address uncertainties, and offer a clearer understanding of the long-term benefits of personalised medicine in psychiatry. Additional funding could also facilitate clinical trials that assess the impact of PGx-guided strategy on medication adherence, patient satisfaction, and overall quality of life. These factors are important for understanding the value of integrating PGx testing into clinical practice. Moreover, as healthcare systems increasingly focus on personalised treatment, there is a strong case for supporting research that investigates the broader implications of PGx-guided strategy beyond just cost savings. This includes its potential to reduce the burden of treatment-resistant depression and improve the overall efficiency of healthcare systems.

It is crucial to acknowledge the extensive variability in CYP2D6 alleles, with over 100 reported to influence phenotype, and their prevalence varies across ethnic groups (Bradford et al., 2002). This diversity can lead to variations in genotype-phenotype relationships between populations, potentially improved with broader allele inclusion, though this may raise costs and warrant further economic assessment. Additionally, disparities in methods for analysing CYP2D6 and CYP2C19 genes among countries and laboratories may impact test accuracy, necessitating consideration when applying findings to other regions along with other factors such as cost.

A major strength of our study is that we utilised a multi-gene analysis. This approach has the potential to enhance the predictive ability of psychiatric pharmacogenomics. Altar et al. conducted a study comparing outcome predictions for subjects using multiple allelic variations in genes, with outcome predictions derived from traditional single-gene analysis. The results showed that multi-gene analysis achieved better discrimination and prediction of outcomes compared to single-gene analysis. This improved clinical validity was attributed to the aggregation of effect sizes for all relevant alleles and the correlation of the composite phenotype with anticipated responses for each psychiatric medication (Altar et al., 2015). Another notable strength of our study is the inclusion of both CYP2D6 and CYP2C19 genes, considering unique patient characteristics such as metabolizer phenotypes. This allows for direct relevance to the clinical populations observed in Qatar. Additionally, our model is drug-specific, incorporating most antidepressants affected by either CYP2D6 or CYP2C19 in Qatar and, consequently, it considers the impact of PGx testing not only on the risk of response and relapse but also on the process of medication selection. Therefore, the model can predict prescription volumes for each medication under different policy strategies. Furthermore, the lifetime horizon of our model is a significant strength as it captures downstream impacts, particularly in terms of subsequent and more resource-intensive treatments.

However, there are several limitations to acknowledge in our study. Firstly, our model was based on the assumption that all patients would be on monotherapy. Additionally, due to the lack of data, our model did not follow up post-relapse depression, underestimating the true benefit of PGx-guided testing. Also not available was data on the direct impact of PGx-guided testing on the probability of recurrence, failing to capture longer-lasting effects on recurrence and underestimating benefits. Moreover, our study focused solely on establishing the cost-effectiveness of PGx-guided testing for MDD in a Qatari context. We did not model the implementation process and the necessary transition to operationalise PGx-guided testing within the Qatari healthcare system. For instance, we did not incorporate the costs associated with establishing the infrastructure required for PGx-guided testing implementation. However, even if additional implementation process costs are to be accounted for, there is no reason to assume that the PGx-guided treatment would not be cost saving. Furthermore, it is important to note that our objective was not to explore the clinical utility of PGx testing, which would require detailed real-world data on the use, such as evidence of clinical validity, sensitivity, specificity, and reliability. Also, to acknowledge, is that we assumed no delayed effect due to genetic testing and subsequent treatment adjustments. In settings where genetic information are not immediately available, especially in primary care, the cost savings will might be overestimated. In addition, evidence-based psychotherapy, such as cognitive–behavioral therapy, has been shown to provide long-term benefits to MDD patients (Brunoni et al., 2014; Cuijpers et al., 2013), particularly when used in conjunction with PGx-guided approaches. Although beyond the scope of our analysis, future research could consider psychotherapy as an adjunct to PGx-guided testing and evaluate the cost-effectiveness of these combined strategies for managing MDD patients. In addition, while patients’ genetic characteristics should be taken into account, it is important to recognise that other diagnostic tools, such as therapeutic drug monitoring and consideration of psychosocial factors, remain crucial for optimising treatment. Lastly, it is possible that response in our Markov model may decline slightly overtime due to relapse in responders. To minimise the impact of these changes, we conducted a scenario analysis that applied a half-cycle correction to avoid overestimating the treatment effect.

## Conclusion

Our findings suggest that the PGx-guided is a valuable evidence-based approach towards filling gaps in practice and addressing uncertainties surrounding the MDD treatment. Implementing the PGx testing to guide antidepressant prescription in Qatar seems to improve health outcomes, while also reducing health system costs.

## Ethics approval

This study was based on literature and available data. Therefore, no ethics approval is required.

## Supplementary Material

Supplemental Material

## Data Availability

Available from the corresponding author upon reasonable request.
