# Supplementary material for: Cost-effectiveness analysis of genotype-guided optimization of major depression treatment in Qatar
Source: J Pharm Policy Pract. 2024 Oct 25;17(1):2410197. doi: 10.1080/20523211.2024.2410197 (PMC11514395; doi:10.1080/20523211.2024.2410197)
Supplement: Supplemental Material [file JPPP_A_2410197_SM7178.docx]

**Supplementary Materials**

**Title**

Cost-effectiveness analysis of genotype-guided optimization of major depression treatment in Qatar

**Table of contents**

| Table S1. Model assumptions, sources, and definitions |  |
| --- | --- |
| Table S2. Short-term probabilities |  |
| Table S3. Transition probabilities  Table S4. Resource utilization  Table S5. Cost-adaptation method |  |
| Table S6. CHEERs Checklist |  |
| Table S7. Validation tools |  |
|  |  |

**Table S1.** Model assumptions, sources, and definitions.

| **Baseline population** |
| --- |
| The model simulated a hypothetical population of 15,000 individuals that reflects the patient characteristics included in the Bradley et al. study (1), where no significant differences in any of the patient baseline characteristics were identified between the study strategy groups. |
| **Cytochrome P450 (CYP) 2D6 and CYP2C19 prevalence** |
| Derived from a 2022 population study of clinically actionable genetic variation affecting drug response from Qatar, analysing 6,045 whole genomes (2). |
| **Outcome probabilities and sources of key model inputs** |
| ***PGx and SoC (CYP2D6, allele carrier and allele non-carrier):***  ***Probabilities of response and relapse were determined based on the following studies:***   - Rush et al. 2006 (STAR*D trial) (3): This study examined the treatment outcomes of four successive steps in the Sequenced Treatment Alternatives to Relieve Depression (STARD) trial in the United States. The trial included adult outpatients with nonpsychotic major depressive disorder (MDD) who underwent one (N=3,671) to four (N=123) acute treatment steps. Patients who did not achieve remission or could not tolerate a treatment step were encouraged to move to the next step. Patients who showed acceptable benefit, preferably symptom remission, in any step could enter a 12-month naturalistic follow-up phase. Remission was defined as a score of ≤5 on the Quick Inventory of Depressive Symptomatology-Self-Report (QIDS-SR (16)) (equivalent to ≤7 on the 17-item Hamilton Rating Scale for Depression [HRSD (17)]), while relapse was defined as a QIDS-SR (16) total score of ≥11 (HRSD (17) ≥14). - Sim et al. 2015 (4): This systematic review and meta-analysis analyzed 97 controlled trials that focused on preventing early relapses or later recurrences in adults diagnosed with MDD. - Sluiter et al. 2019 (5): This study is a cost-utility of pre-emptive genetic testing to support pharmacotherapy in primary care patients with MDD. The Markov model compared the strategy of screening for CYP2D6 and adjusting antidepressant treatment based on a patient's metabolizer profile (poor, extensive, or ultra-metabolizer) with the strategy of no screening at week 6 of initiation treatment. The study was conducted from the societal perspective in the Netherlands.   ***PGx and SoC (CYP2C19, allele carrier and allele non-carrier):***  ***Probabilities of response and relapse were determined based on the following studies:***   - Carta et al. 2022 (6): This study conducted a cost-utility analysis to assess the screening of CYP2C19 and CYP2D6 in patients with MDD. The aim was to identify the main drivers influencing cost-utility. Two Markov models were developed from an Italian societal perspective, considering pharmacogenetic testing to characterize the metabolizing profiles of CYP2C19 and CYP2D6 in a hypothetical case study of patients with MDD. The models explored different scenarios of antidepressant treatment adjustment based on the patient's metabolizing profile or treatment over an 18-week period. - Sim et al. 2015 (4): as described previously. - Fabbri et al. 2021 (7): This study conducted a cost-effectiveness analysis to compare genetic and clinical predictors for selecting combined psychotherapy and pharmacotherapy in MDD with standard care (pharmacotherapy for all subjects) over a three-year period. The analysis utilized a Markov model from the perspective of the United Kingdom (UK) National Health System. - Rush et al. 2006 (STAR*D trial) (3): as described previously.   ***PGx and SoC (CYP2D6, allele carrier and allele non-carrier):***  ***Probabilities of side effect were determined based on the following studies:***   - Shams et al. 2006 (8): This study aimed to assess the impact of the O-demethylation phenotype of venlafaxine on pharmacokinetics and clinical outcomes. In a sample of 100 patients treated with venlafaxine, serum concentrations of venlafaxine, desmethylvenlafaxine, and N-desmethylvenlafaxine, as well as the ratios of desmethylvenlafaxine/venlafaxine concentrations as a measure of O-demethylation, were determined. Patients exhibiting abnormally high or low metabolic ratios were selected for genotyping. Clinical effects were monitored using the Clinical Global Impressions Scale, and side effects were assessed using the Udvalg for Kliniske Undersogelser Side Effect Rating Scale.   ***PGx and SoC (CYP2C19, allele carrier and allele non-carrier):***  ***Probabilities of side effect were determined based on the following studies:***   - Carta et al. 2022 (6): as described previously. - Shams et al. 2006 (8): as described previously. - Fabbri et al. 2021 (7): as described previously.   ***PGx and SoC (CYP2D6 and CYP2C19, allele carrier and allele non-carrier):***  ***Probabilities of switching to alternative, titrating dose, observation, discontinuation, suicide attempt, suicide death were determined based on the following studies:***   - Bradley et al. 2018 (1): This study aimed to evaluate the effect of pharmacogenetics-guided treatment on patients diagnosed with depression and/or anxiety, comparing it to the SoC. The trial followed a prospective, randomized, subject- and rater-blinded design and enrolled 685 patients. The NeuroIDgenetix® test was utilized, which utilized a genetic variant panel of ten genes in conjunction with concomitant medications to provide medication management recommendations based on gene-drug and drug-drug interactions for over 40 medications commonly used in the treatment of depression and anxiety. Pharmacogenetic testing was conducted during the initial screening visit, and baseline patient assessments were determined using the HAM-D17 and the HAM-A. Following enrollment and randomization, pharmacogenetic results for subjects assigned to the experimental group were shared with physicians to guide treatment selection, while control subjects received treatment according to the standard of care. Assessments using HAM-D17 and HAM-A were collected at 4 weeks, 8 weeks, and 12 weeks after baseline to evaluate the efficacy of therapeutic selection. |
| **Utility data** |
| Utility of response:   - Sullivan et. al. 2006 (9): This study aimed to determine age- and sex-specific utilities for chronic conditions in the United States, including depression, using the Preference-Based EQ-5D Index Scores. The researchers utilized a nationally representative dataset of 38,678 adults obtained from the Medical Expenditure Panel Survey (2000-2002).   Utility of relapse and no response:   - Kuyken et al. 2008 (10): In this study, a Preference-Based EQ-5D Index Scores was used to assess utilities among individuals at risk of depressive relapse who were treated with antidepressant medication (ADM). The aim was to compare the utility outcomes of mindfulness-based cognitive therapy (MBCT) with those of maintenance ADM (m-ADM). The study followed a parallel 2-group randomized controlled trial design, with one group receiving m-ADM (N = 62) and the other group receiving MBCT plus support for tapering/discontinuing antidepressants (N = 61). |
| **Markov model health states** |
| Derived from our short-term decision-tree analysis. |
| **Non-mental health mortality in Markov model** |
| Calculated based on mental health mortality and all-cause mortality derived from the Births & Deaths reports (latest available) (11). |
| **Panel genetic screening** |
| For the base-case analysis, the model assumed 100% specificity, sensitivity, and uptake. |
| **Patients treated with** **antidepressants** |
| According to CIPC 2023 (12), paroxetine, vortioxetine, fluvoxamine, and venlafaxine are strongly associated with CYP2D6, while escitalopram and sertraline have the strongest effect on CYP2C19. Based on data from Mental Health, Qatar (13). the model assumed that 16% of patients would receive CYP2D6 medications and 84% would receive CYP2C19 medications. |

**Table S2.** Short-term model probabilities.

| **Panel PGx group** | | |
| --- | --- | --- |
| **CYP2D6 carrier** | | |
| **Poor metabolizer** | | |
| **Response without side effects** | Relapse required switching to alternative | 0.01992672 |
|  | Relapse required observing/waiting | 0.00732600 |
|  | Relapse required discontinuation | 0.00761904 |
|  | Relapse led to suicide death | 0.00001108 |
|  | Relapse led to suicide survival | 0.00174716 |
|  | No relapse | 0.33337000 |
| **Response with side effects** | Relapse required switching to alternative | 0.00976409 |
|  | Relapse required observing/waiting | 0.00358974 |
|  | Relapse required discontinuation | 0.00373333 |
|  | Relapse led to suicide death | 0.00000543 |
|  | Relapse led to suicide survival | 0.00085611 |
|  | No relapse | 0.163351 |
| **No response** | Switching to alternative | 0.20341067 |
|  | Increasing dose | 0.07478333 |
|  | Observing/waiting | 0.07478333 |
|  | Discontinuation | 0.07777467 |
|  | Suicide death | 0.00011307 |
|  | Suicide survival | 0.01783493 |

| **Intermediate metabolizer** | | |
| --- | --- | --- |
| **Response without side effects** | Relapse required switching to alternative | 0.019927 |
|  | Relapse required observing/waiting | 0.007326 |
|  | Relapse required discontinuation | 0.007619 |
|  | Relapse led to suicide death | 0.000011 |
|  | Relapse led to suicide survival | 0.001747 |
|  | No relapse | 0.333370 |
| **Response with side effects** | Relapse required switching to alternative | 0.009764 |
|  | Relapse required observing/waiting | 0.003590 |
|  | Relapse required discontinuation | 0.003733 |
|  | Relapse led to suicide death | 0.000005 |
|  | Relapse led to suicide survival | 0.000856 |
|  | No relapse | 0.163351 |
| **No response** | Switching to alternative | 0.203411 |
|  | Increasing dose | 0.074783 |
|  | Observing/waiting | 0.074783 |
|  | Discontinuation | 0.077775 |
|  | Suicide death | 0.000113 |
|  | Suicide survival | 0.017835 |

| **Ultra-rapid metabolizer** | | |
| --- | --- | --- |
| **Response without side effects** | Relapse required switching to alternative | 0.007315 |
|  | Relapse required observing/waiting | 0.002689 |
|  | Relapse required discontinuation | 0.026636 |
|  | Relapse led to suicide death | 0.000004 |
|  | Relapse led to suicide survival | 0.000641 |
|  | No relapse | 0.122371 |
| **Response with side effects** | Relapse required switching to alternative | 0.00731456 |
|  | Relapse required observing/waiting | 0.00268918 |
|  | Relapse required discontinuation | 0.00279674 |
|  | Relapse led to suicide death | 0.00000407 |
|  | Relapse led to suicide survival | 0.00064134 |
|  | No relapse | 0.12237107 |
| **No response** | Switching to alternative | 0.319579 |
|  | Increasing dose | 0.117492 |
|  | Observing/waiting | 0.117492 |
|  | Discontinuation | 0.122192 |
|  | Suicide death | 0.000178 |
|  | Suicide survival | 0.028021 |

| **Panel PGx group** | | |
| --- | --- | --- |
| **CYP2D6 non-carrier** | | |
| **Poor metabolizer** | | |
| **Response without side effects** | Relapse required switching to alternative | 0.019923 |
|  | Relapse required observing/waiting | 0.007325 |
|  | Relapse required discontinuation | 0.007618 |
|  | Relapse led to suicide death | 0.000011 |
|  | Relapse led to suicide survival | 0.001747 |
|  | No relapse | 0.333307 |
| **Response with side effects** | Relapse required switching to alternative | 0.009762 |
|  | Relapse required observing/waiting | 0.003589 |
|  | Relapse required discontinuation | 0.003733 |
|  | Relapse led to suicide death | 0.000005 |
|  | Relapse led to suicide survival | 0.000856 |
|  | No relapse | 0.163320 |
| **No response** | Switching to alternative | 0.203372 |
|  | Increasing dose | 0.074769 |
|  | Observing/waiting | 0.074769 |
|  | Discontinuation | 0.077760 |
|  | Suicide death | 0.000113 |
|  | Suicide survival | 0.017832 |

| **Intermediate metabolizer** | | |
| --- | --- | --- |
| **Response without side effects** | Relapse required switching to alternative | 0.019880 |
|  | Relapse required observing/waiting | 0.007309 |
|  | Relapse required discontinuation | 0.007601 |
|  | Relapse led to suicide death | 0.000011 |
|  | Relapse led to suicide survival | 0.001743 |
|  | No relapse | 0.332594 |
| **Response with side effects** | Relapse required switching to alternative | 0.009741 |
|  | Relapse required observing/waiting | 0.003581 |
|  | Relapse required discontinuation | 0.003725 |
|  | Relapse led to suicide death | 0.000005 |
|  | Relapse led to suicide survival | 0.000854 |
|  | No relapse | 0.162971 |
| **No response** | Switching to alternative | 0.202937 |
|  | Increasing dose | 0.074609 |
|  | Observing/waiting | 0.074609 |
|  | Discontinuation | 0.077594 |
|  | Suicide death | 0.000113 |
|  | Suicide survival | 0.017793 |

| **Ultra-rapid metabolizer** | | |
| --- | --- | --- |
| **Response without side effects** | Relapse required switching to alternative | 0.018833 |
|  | Relapse required observing/waiting | 0.006924 |
|  | Relapse required discontinuation | 0.007201 |
|  | Relapse led to suicide death | 0.000010 |
|  | Relapse led to suicide survival | 0.001651 |
|  | No relapse | 0.315080 |
| **Response with side effects** | Relapse required switching to alternative | 0.005650 |
|  | Relapse required observing/waiting | 0.002077 |
|  | Relapse required discontinuation | 0.002160 |
|  | Relapse led to suicide death | 0.000003 |
|  | Relapse led to suicide survival | 0.000495 |
|  | No relapse | 0.094524 |
| **No response** | Switching to alternative | 0.246855 |
|  | Increasing dose | 0.090756 |
|  | Observing/waiting | 0.090756 |
|  | Discontinuation | 0.094386 |
|  | Suicide death | 0.000137 |
|  | Suicide survival | 0.021644 |

| **SoC group** | | |
| --- | --- | --- |
| **CYP2D6 carrier** | | |
| **Poor metabolizer** | | |
| **Response without side effects** | Relapse required switching to alternative | 0.046184 |
|  | Relapse required observing/waiting | 0.025658 |
|  | Relapse required discontinuation | 0.008210 |
|  | Relapse led to suicide death | 0.000039 |
|  | Relapse led to suicide survival | 0.006119 |
|  | No relapse | 0.283790 |
| **Response with side effects** | Relapse required switching to alternative | 0.041566 |
|  | Relapse required observing/waiting | 0.023092 |
|  | Relapse required discontinuation | 0.007389 |
|  | Relapse led to suicide death | 0.000000 |
|  | Relapse led to suicide survival | 0.005507 |
|  | No relapse | 0.255411 |
| **No response** | Switching to alternative | 0.128312 |
|  | Increasing dose | 0.057484 |
|  | Observing/waiting | 0.071285 |
|  | Discontinuation | 0.022811 |
|  | Suicide death | 0.000108 |
|  | Suicide survival | 0.017001 |

| **Intermediate metabolizer** | | |
| --- | --- | --- |
| **Response without side effects** | Relapse required switching to alternative | 0.046184 |
|  | Relapse required observing/waiting | 0.025658 |
|  | Relapse required discontinuation | 0.008210 |
|  | Relapse led to suicide death | 0.000039 |
|  | Relapse led to suicide survival | 0.006119 |
|  | No relapse | 0.283790 |
| **Response with side effects** | Relapse required switching to alternative | 0.032329 |
|  | Relapse required observing/waiting | 0.017960 |
|  | Relapse required discontinuation | 0.005747 |
|  | Relapse led to suicide death | 0.000027 |
|  | Relapse led to suicide survival | 0.004283 |
|  | No relapse | 0.198653 |
| **No response** | Switching to alternative | 0.160282 |
|  | Increasing dose | 0.071806 |
|  | Observing/waiting | 0.089046 |
|  | Discontinuation | 0.028495 |
|  | Suicide death | 0.000135 |
|  | Suicide survival | 0.021236 |

| **Ultra-rapid metabolizer** | | |
| --- | --- | --- |
| **Response without side effects** | Relapse required switching to alternative | 0.043688 |
|  | Relapse required observing/waiting | 0.024271 |
|  | Relapse required discontinuation | 0.007767 |
|  | Relapse led to suicide death | 0.000037 |
|  | Relapse led to suicide survival | 0.005788 |
|  | No relapse | 0.268450 |
| **Response with side effects** | Relapse required switching to alternative | 0.013106 |
|  | Relapse required observing/waiting | 0.007281 |
|  | Relapse required discontinuation | 0.002330 |
|  | Relapse led to suicide death | 0.000011 |
|  | Relapse led to suicide survival | 0.001736 |
|  | No relapse | 0.080535 |
| **No response** | Switching to alternative | 0.235455 |
|  | Increasing dose | 0.105484 |
|  | Observing/waiting | 0.130808 |
|  | Discontinuation | 0.041859 |
|  | Suicide death | 0.000198 |
|  | Suicide survival | 0.031196 |

| **SoC group** | | |
| --- | --- | --- |
| **CYP2D6 non-carrier** | | |
| **Poor metabolizer** | | |
| **Response without side effects** | Relapse required switching to alternative | 0.046184 |
|  | Relapse required observing/waiting | 0.025658 |
|  | Relapse required discontinuation | 0.008210 |
|  | Relapse led to suicide death | 0.000039 |
|  | Relapse led to suicide survival | 0.006119 |
|  | No relapse | 0.283790 |
| **Response with side effects** | Relapse required switching to alternative | 0.041566 |
|  | Relapse required observing/waiting | 0.023092 |
|  | Relapse required discontinuation | 0.007389 |
|  | Relapse led to suicide death | 0.000035 |
|  | Relapse led to suicide survival | 0.005507 |
|  | No relapse | 0.255411 |
| **No response** | Switching to alternative | 0.128312 |
|  | Increasing dose | 0.057484 |
|  | Observing/waiting | 0.071285 |
|  | Discontinuation | 0.022811 |
|  | Suicide death | 0.000108 |
|  | Suicide survival | 0.017001 |

| **Intermediate metabolizer** | | |
| --- | --- | --- |
| **Response without side effects** | Relapse required switching to alternative | 0.046184 |
|  | Relapse required observing/waiting | 0.025658 |
|  | Relapse required discontinuation | 0.008210 |
|  | Relapse led to suicide death | 0.000039 |
|  | Relapse led to suicide survival | 0.006119 |
|  | No relapse | 0.283790 |
| **Response with SE** | Relapse required switching to alternative | 0.032329 |
|  | Relapse required observing/waiting | 0.017960 |
|  | Relapse required discontinuation | 0.005747 |
|  | Relapse led to suicide death | 0.000027 |
|  | Relapse led to suicide survival | 0.004283 |
|  | No relapse | 0.198653 |
| **No response** | Switching to alternative | 0.160282 |
|  | Increasing dose | 0.071806 |
|  | Observing/waiting | 0.089046 |
|  | Discontinuation | 0.028495 |
|  | Suicide death | 0.000135 |
|  | Suicide survival | 0.021236 |

| **Ultra-rapid metabolizer** | | |
| --- | --- | --- |
| **Response without side effects** | Relapse required switching to alternative | 0.043688 |
|  | Relapse required observing/waiting | 0.024271 |
|  | Relapse required discontinuation | 0.007767 |
|  | Relapse led to suicide death | 0.000037 |
|  | Relapse led to suicide survival | 0.005788 |
|  | No relapse | 0.268450 |
| **Response with side effects** | Relapse required switching to alternative | 0.013106 |
|  | Relapse required observing/waiting | 0.007281 |
|  | Relapse required discontinuation | 0.002330 |
|  | Relapse led to suicide death | 0.000011 |
|  | Relapse led to suicide survival | 0.001736 |
|  | No relapse | 0.080535 |
| **No response** | Switching to alternative | 0.000000 |
|  | Increasing dose | 0.186435 |
|  | Observing/waiting | 0.231195 |
|  | Discontinuation | 0.073982 |
|  | Suicide death | 0.000350 |
|  | Suicide survival | 0.055137 |

| **Panel PGx** | | |
| --- | --- | --- |
| **CYP2C19 carrier** | | |
| **Poor metabolizer** | | |
| **Response without side effects** | Relapse required switching to alternative | 0.03123648 |
|  | Relapse required observing/waiting | 0.01148400 |
|  | Relapse required discontinuation | 0.01194336 |
|  | Relapse led to suicide death | 0.00001736 |
|  | Relapse led to suicide survival | 0.00273880 |
| **Success** | No relapse | 0.52258000 |
| **Response with side effects** | Relapse required switching to alternative | 0.01530588 |
|  | Relapse required observing/waiting | 0.00562716 |
|  | Relapse required discontinuation | 0.00585225 |
|  | Relapse led to suicide death | 0.00000851 |
|  | Relapse led to suicide survival | 0.00134201 |
| **Success** | No relapse | 0.256064 |
| **No response** | Switching to alternative | 0.06156267 |
|  | Increasing dose | 0.02263333 |
|  | Observing/waiting | 0.02263333 |
|  | Discontinuation | 0.02353867 |
|  | Suicide death | 0.00003422 |
|  | Suicide survival | 0.00539778 |

| **Intermediate metabolizer** | | |
| --- | --- | --- |
| **Response without side effects** | Relapse required switching to alternative | 0.025851 |
|  | Relapse required observing/waiting | 0.009504 |
|  | Relapse required discontinuation | 0.009884 |
|  | Relapse led to suicide death | 0.000014 |
|  | Relapse led to suicide survival | 0.002267 |
|  | No relapse | 0.432480 |
| **Response with side effects** | Relapse required switching to alternative | 0.012667 |
|  | Relapse required observing/waiting | 0.004657 |
|  | Relapse required discontinuation | 0.004843 |
|  | Relapse led to suicide death | 0.000007 |
|  | Relapse led to suicide survival | 0.001111 |
|  | No relapse | 0.211915 |
| **No response** | Switching to alternative | 0.129109 |
|  | Increasing dose | 0.047467 |
|  | Observing/waiting | 0.047467 |
|  | Discontinuation | 0.049365 |
|  | Suicide death | 0.000072 |
|  | Suicide survival | 0.011320 |

| **Ultra-rapid metabolizer** | | |
| --- | --- | --- |
| **Response without side effects** | Relapse required switching to alternative | 0.019927 |
|  | Relapse required observing/waiting | 0.007326 |
|  | Relapse required discontinuation | 0.007619 |
|  | Relapse led to suicide death | 0.000011 |
|  | Relapse led to suicide survival | 0.001747 |
|  | No relapse | 0.333370 |
| **Response with side effects** | Relapse required switching to alternative | 0.00976409 |
|  | Relapse required observing/waiting | 0.00358974 |
|  | Relapse required discontinuation | 0.00373333 |
|  | Relapse led to suicide death | 0.00000543 |
|  | Relapse led to suicide survival | 0.00085611 |
|  | No relapse | 0.16335130 |
| **No response** | Switching to alternative | 0.203411 |
|  | Increasing dose | 0.074783 |
|  | Observing/waiting | 0.074783 |
|  | Discontinuation | 0.077775 |
|  | Suicide death | 0.000113 |
|  | Suicide survival | 0.017835 |

| **Rapid metabolizer** | | |
| --- | --- | --- |
| **Response without side effects** | Relapse required switching to alternative | 0.019927 |
|  | Relapse required observing/waiting | 0.007326 |
|  | Relapse required discontinuation | 0.007619 |
|  | Relapse led to suicide death | 0.000011 |
|  | Relapse led to suicide survival | 0.001747 |
|  | No relapse | 0.333370 |
| **Response with side effects** | Relapse required switching to alternative | 0.009764 |
|  | Relapse required observing/waiting | 0.003590 |
|  | Relapse required discontinuation | 0.003733 |
|  | Relapse led to suicide death | 0.000005 |
|  | Relapse led to suicide survival | 0.000856 |
|  | No relapse | 0.163351 |
| **No response** | Switching to alternative | 0.203411 |
|  | Increasing dose | 0.074783 |
|  | Observing/waiting | 0.074783 |
|  | Discontinuation | 0.077775 |
|  | Suicide death | 0.000113 |
|  | Suicide survival | 0.017835 |

| **Panel PGx** | | | |
| --- | --- | --- | --- |
| **CYP2C19 non-carrier** | | | |
| **Poor metabolizer** | | | |
| **Response without side effects** | Relapse required switching to alternative | | 0.031231 |
|  | Relapse required observing/waiting | | 0.011482 |
|  | Relapse required discontinuation | | 0.011941 |
|  | Relapse led to suicide death | | 0.000017 |
|  | Relapse led to suicide survival | | 0.002738 |
|  | No relapse | | 0.522482 |
| **Response with side effects** | Relapse required switching to alternative | | 0.015303 |
|  | Relapse required observing/waiting | | 0.005626 |
|  | Relapse required discontinuation | | 0.005851 |
|  | Relapse led to suicide death | | 0.000009 |
|  | Relapse led to suicide survival | | 0.001342 |
|  | No relapse | | 0.256016 |
| **No response** | Switching to alternative | | 0.061551 |
|  | Increasing dose | | 0.022629 |
|  | Observing/waiting | | 0.022629 |
|  | Discontinuation | | 0.023534 |
|  | Suicide death | | 0.000034 |
|  | Suicide survival | | 0.005397 |
| **Intermediate metabolizer** | | | |
| **Response without side effects** | Relapse required switching to alternative | 0.025800 | |
|  | Relapse required observing/waiting | 0.009485 | |
|  | Relapse required discontinuation | 0.009865 | |
|  | Relapse led to suicide death | 0.000014 | |
|  | Relapse led to suicide survival | 0.002262 | |
|  | No relapse | 0.431624 | |
| **Response with side effects** | Relapse required switching to alternative | 0.012642 | |
|  | Relapse required observing/waiting | 0.004648 | |
|  | Relapse required discontinuation | 0.004834 | |
|  | Relapse led to suicide death | 0.000007 | |
|  | Relapse led to suicide survival | 0.001108 | |
|  | No relapse | 0.211496 | |
| **No response** | Switching to alternative | 0.128854 | |
|  | Increasing dose | 0.047373 | |
|  | Observing/waiting | 0.047373 | |
|  | Discontinuation | 0.049268 | |
|  | Suicide death | 0.000072 | |
|  | Suicide survival | 0.011298 | |

| **Ultra-rapid metabolizer** | | |
| --- | --- | --- |
| **Response without side effects** | Relapse required switching to alternative | 0.019914 |
|  | Relapse required observing/waiting | 0.007321 |
|  | Relapse required discontinuation | 0.007614 |
|  | Relapse led to suicide death | 0.000011 |
|  | Relapse led to suicide survival | 0.001746 |
|  | No relapse | 0.333152 |
| **Response with SE** | Relapse required switching to alternative | 0.009758 |
|  | Relapse required observing/waiting | 0.003587 |
|  | Relapse required discontinuation | 0.003731 |
|  | Relapse led to suicide death | 0.000005 |
|  | Relapse led to suicide survival | 0.000856 |
|  | No relapse | 0.163245 |
| **No response** | Switching to alternative | 0.203278 |
|  | Increasing dose | 0.074734 |
|  | Observing/waiting | 0.074734 |
|  | Discontinuation | 0.077724 |
|  | Suicide death | 0.000113 |
|  | Suicide survival | 0.017823 |

| **Rapid metabolizer** | | |
| --- | --- | --- |
| **Response without side effects** | Relapse required switching to alternative | 0.016868 |
|  | Relapse required observing/waiting | 0.006202 |
|  | Relapse required discontinuation | 0.006450 |
|  | Relapse led to suicide death | 0.000009 |
|  | Relapse led to suicide survival | 0.001479 |
|  | No relapse | 0.282206 |
| **Response with side effects** | Relapse required switching to alternative | 0.008266 |
|  | Relapse required observing/waiting | 0.003039 |
|  | Relapse required discontinuation | 0.003160 |
|  | Relapse led to suicide death | 0.000005 |
|  | Relapse led to suicide survival | 0.000725 |
|  | No relapse | 0.138281 |
| **No response** | Switching to alternative | 0.241768 |
|  | Increasing dose | 0.088885 |
|  | Observing/waiting | 0.088885 |
|  | Discontinuation | 0.092441 |
|  | Suicide death | 0.000134 |
|  | Suicide survival | 0.021198 |

| **SoC group** | | |
| --- | --- | --- |
| **CYP2C19 carrier** | | |
| **Poor metabolizer** | | |
| **Response without side effects** | Relapse required switching to alternative | 0.059914 |
|  | Relapse required observing/waiting | 0.033286 |
|  | Relapse required discontinuation | 0.010651 |
|  | Relapse led to suicide death | 0.000050 |
|  | Relapse led to suicide survival | 0.007938 |
|  | No relapse | 0.368160 |
| **Response with side effects** | Relapse required switching to alternative | 0.032953 |
|  | Relapse required observing/waiting | 0.018307 |
|  | Relapse required discontinuation | 0.005858 |
|  | Relapse led to suicide death | 0.000000 |
|  | Relapse led to suicide survival | 0.004366 |
|  | No relapse | 0.202488 |
| **No response** | Switching to alternative | 0.116053 |
|  | Increasing dose | 0.042667 |
|  | Observing/waiting | 0.042667 |
|  | Discontinuation | 0.044373 |
|  | Suicide death | 0.000065 |
|  | Suicide survival | 0.010175 |

| **Intermediate metabolizer** | | |
| --- | --- | --- |
| **Response without side effects** | Relapse required switching to alternative | 0.046184 |
|  | Relapse required observing/waiting | 0.025658 |
|  | Relapse required discontinuation | 0.008210 |
|  | Relapse led to suicide death | 0.000039 |
|  | Relapse led to suicide survival | 0.006119 |
|  | No relapse | 0.283790 |
| **Response with side effects** | Relapse required switching to alternative | 0.023554 |
|  | Relapse required observing/waiting | 0.013085 |
|  | Relapse required discontinuation | 0.004187 |
|  | Relapse led to suicide death | 0.000020 |
|  | Relapse led to suicide survival | 0.003121 |
|  | No relapse | 0.144733 |
| **No response** | Switching to alternative | 0.200056 |
|  | Increasing dose | 0.073550 |
|  | Observing/waiting | 0.073550 |
|  | Discontinuation | 0.076492 |
|  | Suicide death | 0.000111 |
|  | Suicide survival | 0.017541 |

| **Ultra-rapid metabolizer** | | |
| --- | --- | --- |
| **Response without side effects** | Relapse required switching to alternative | 0.046184 |
|  | Relapse required observing/waiting | 0.025658 |
|  | Relapse required discontinuation | 0.008210 |
|  | Relapse led to suicide death | 0.000039 |
|  | Relapse led to suicide survival | 0.006119 |
|  | No relapse | 0.283790 |
| **Response with side effects** | Relapse required switching to alternative | 0.022630 |
|  | Relapse required observing/waiting | 0.012572 |
|  | Relapse required discontinuation | 0.004023 |
|  | Relapse led to suicide death | 0.000019 |
|  | Relapse led to suicide survival | 0.002998 |
|  | No relapse | 0.139057 |
| **No response** | Switching to alternative | 0.203411 |
|  | Increasing dose | 0.074783 |
|  | Observing/waiting | 0.074783 |
|  | Discontinuation | 0.077775 |
|  | Suicide death | 0.000113 |
|  | Suicide survival | 0.017835 |

| **Rapid metabolizer** | | |
| --- | --- | --- |
| **Response without side effects** | Relapse required switching to alternative | 0.046184 |
|  | Relapse required observing/waiting | 0.025658 |
|  | Relapse required discontinuation | 0.008210 |
|  | Relapse led to suicide death | 0.000039 |
|  | Relapse led to suicide survival | 0.006119 |
|  | No relapse | 0.283790 |
| **Response with side effects** | Relapse required switching to alternative | 0.041566 |
|  | Relapse required observing/waiting | 0.023092 |
|  | Relapse required discontinuation | 0.007389 |
|  | Relapse led to suicide death | 0.000035 |
|  | Relapse led to suicide survival | 0.005507 |
|  | No relapse | 0.255411 |
| **No response** | Switching to alternative | 0.134640 |
|  | Increasing dose | 0.049500 |
|  | Observing/waiting | 0.049500 |
|  | Discontinuation | 0.051480 |
|  | Suicide death | 0.000075 |
|  | Suicide survival | 0.011805 |

| **SoC group** | | |
| --- | --- | --- |
| **CYP2C19 non-carrier** | | |
| **Poor metabolizer** | | |
| **Response without side effects** | Relapse required switching to alternative | 0.059914 |
|  | Relapse required observing/waiting | 0.033286 |
|  | Relapse required discontinuation | 0.010651 |
|  | Relapse led to suicide death | 0.000050 |
|  | Relapse led to suicide survival | 0.007938 |
|  | No relapse | 0.368160 |
| **Response with side effects** | Relapse required switching to alternative | 0.032953 |
|  | Relapse required observing/waiting | 0.018307 |
|  | Relapse required discontinuation | 0.005858 |
|  | Relapse led to suicide death | 0.000028 |
|  | Relapse led to suicide survival | 0.004366 |
|  | No relapse | 0.202488 |
| **No response** | Switching to alternative | 0.116053 |
|  | Increasing dose | 0.042667 |
|  | Observing/waiting | 0.042667 |
|  | Discontinuation | 0.044373 |
|  | Suicide death | 0.000065 |
|  | Suicide survival | 0.010175 |

| **Intermediate metabolizer** | | |
| --- | --- | --- |
| **Response without side effects** | Relapse required switching to alternative | 0.046184 |
|  | Relapse required observing/waiting | 0.025658 |
|  | Relapse required discontinuation | 0.008210 |
|  | Relapse led to suicide death | 0.000039 |
|  | Relapse led to suicide survival | 0.006119 |
|  | No relapse | 0.283790 |
| **Response with side effects** | Relapse required switching to alternative | 0.023554 |
|  | Relapse required observing/waiting | 0.013085 |
|  | Relapse required discontinuation | 0.004187 |
|  | Relapse led to suicide death | 0.000020 |
|  | Relapse led to suicide survival | 0.003121 |
|  | No relapse | 0.144733 |
| **No response** | Switching to alternative | 0.200056 |
|  | Increasing dose | 0.073550 |
|  | Observing/waiting | 0.073550 |
|  | Discontinuation | 0.076492 |
|  | Suicide death | 0.000111 |
|  | Suicide survival | 0.017541 |

| **Ultra-rapid metabolizer** | | |
| --- | --- | --- |
| **Response without side effects** | Relapse required switching to alternative | 0.046184 |
|  | Relapse required observing/waiting | 0.025658 |
|  | Relapse required discontinuation | 0.008210 |
|  | Relapse led to suicide death | 0.000039 |
|  | Relapse led to suicide survival | 0.006119 |
|  | No relapse | 0.283790 |
| **Response with side effects** | Relapse required switching to alternative | 0.022630 |
|  | Relapse required observing/waiting | 0.012572 |
|  | Relapse required discontinuation | 0.004023 |
|  | Relapse led to suicide death | 0.000019 |
|  | Relapse led to suicide survival | 0.002998 |
|  | No relapse | 0.139057 |
| **No response** | Switching to alternative | 0.000000 |
|  | Increasing dose | 0.155487 |
|  | Observing/waiting | 0.155487 |
|  | Discontinuation | 0.161706 |
|  | Suicide death | 0.000235 |
|  | Suicide survival | 0.037082 |

| **Rapid metabolizer** | | |
| --- | --- | --- |
| **Response without side effects** | Relapse required switching to alternative | 0.046184 |
|  | Relapse required observing/waiting | 0.025658 |
|  | Relapse required discontinuation | 0.008210 |
|  | Relapse led to suicide death | 0.000039 |
|  | Relapse led to suicide survival | 0.006119 |
|  | No relapse | 0.283790 |
| **Response with side effects** | Relapse required switching to alternative | 0.022630 |
|  | Relapse required observing/waiting | 0.012572 |
|  | Relapse required discontinuation | 0.004023 |
|  | Relapse led to suicide death | 0.000019 |
|  | Relapse led to suicide survival | 0.002998 |
|  | No relapse | 0.139057 |
| **No response** | Switching to alternative | 0.203411 |
|  | Increasing dose | 0.074783 |
|  | Observing/waiting | 0.074783 |
|  | Discontinuation | 0.077775 |
|  | Suicide death | 0.000113 |
|  | Suicide survival | 0.017835 |

**Table S3.** Transition probabilities, as determined from our decision-analytic tree analysis.

- For the health state ‘response with or without side effects and without relapse’, the probability was set at 0.5268 for the PGx testing group and 0.4733 for the SoC at age 41.
- For the health state ‘relapse with or without side effects’, the probability was set at 0.2359 for the PGx testing group and 0.5201 for the SoC at age 41.
- For the health state ‘suicide death with or without side effects and without response’, the probability was set at 0.0008 for the PGx testing group and 0.0013 for the SoC at age 41.
- For the health state ‘non-suicide death’, the probability was set at 0.4060 for both groups at age 41.
- For the health state ‘no response’, the probability was set at 0.9742 for the PGx testing group and 1 for the SoC at age 41.
- For the health states ‘death from suicide causes’ and ‘death from non-suicide causes’, the proportions of deaths were obtained from local Qatari data. The data indicated that 5.8% and 40.6% of deaths were due to suicide and non-suicide causes, respectively (11).

Thus, to calculate the transition probability of an event at aged 41, the functions below were used to transform probabilities into transition probabilities using the formula by Briggs et al. (14):

- *r = (- ln(1−p)) /t, where r is the 3-month rate, p is the proportion of event, and t is duration of study.*
- *tp = 1−e−rt, where r is the 3-month adjusted rate and t is the cycle length.*

MDD risk was age-adjusted using mental health mortality age-related trends in the Qatari population (15,16).

Equations of extrapolation of death data were executed to derive age-specific death rates.

*Where: x = age.*

*Where: x = age.*

**Table S4.** Resource utilization

| **Pharmaceuticals** | | |
| --- | --- | --- |
| **Item** | **Indication** | **Details** |
| Escitalopram | First- and second-line | 10 mg once daily for 6 weeks |
| Paroxetine | First- and second-line | 20 mg once daily for 6 weeks |
| Sertraline | First- and second-line | 50 mg once daily for 6 weeks |
| Vortioxetine | First- and second-line | 10 mg once daily for 6 weeks |
| Venlafaxine | First- and second-line | 75 mg once daily for 6 weeks |
| **Side effects management** | | |
| Transfer to urology clinic | Sexual dysfunction | Once |
| Sodium lab test | Hyponatremia | Once |
| Sodium bicarbonate 8.4% replacement |  | Once |
| Emergency visit |  | Once |
| Electrocardiogram | QT prolongation | Once |
| Emergency visit | Serotonin syndrome |  |
| Lorazepam |  | Solution once |
| Reduction in dose | Bruxism | ------ |
| Trazodone | Insomnia | 50 mg once daily for 30 days |
| **Screening and laboratory** | | |
| Complete blood count | -------- | At baseline, at 6 weeks, and at 3-month follow-up |
| Electrocardiogram |  | At baseline, at 6 weeks, and at 3-month follow-up |
|  |  | At baseline, at 6 weeks, and at 3-month follow-up |
| Creatinine |  | At baseline, at 6 weeks, and at 3-month follow-up |
| Sodium |  | At baseline, at 6 weeks, and at 3-month follow-up |
| Potassium |  | At baseline, at 6 weeks, and at 3-month follow-up |
| Chloride |  | At baseline, at 6 weeks, and at 3-month follow-up |
| Bicarbonate |  | At baseline, at 6 weeks, and at 3-month follow-up |
| Magnesium |  | At baseline, at 6 weeks, and at 3-month follow-up |
| Calcium |  | At baseline, at 6 weeks, and at 3-month follow-up |
| **Events** | | |
| Relapse | -------- | At 6 weeks and at 3-month follow-up |
| Suicide death |  | At 6 weeks and at 3-month follow-up |
| Non-suicide death |  | At 6 weeks and at 3-month follow-up |
| Survival |  | At 6 weeks and at 3-month follow-up |
| **Follow-up** | | |
| Consultation visit |  | At 6 weeks initially then every 3-month |

**Table S5.** Cost-adaptation method

The cost-adaptation process was performed following the method developed and validated by a study (10).

**Step 1. Adjustment for resource utilization (health spending).**

Healthcare expenses per capita from the Organization for Economic Co-operation and Development (OECD) and adjusted for purchasing power were considered. The correction factor between Qatar and the United States (US) and Belgium was calculated by diving the current expenditure on health per capita in Qatar in a specified year by the expenditure on health in the US and Belgium for the same year.

| **Country** | **Expenses per capita** |
| --- | --- |
| **Qatar** | 2,737 |
| **US (2018)** | 1,348 |
| **Belgium (2005)** | 3,104 |

**Step 2. Adjustment for prices of healthcare services.**

The correction for different healthcare prices between Qatar and US was considered through the purchasing power parity. As in step 1, a correction factor was applied.

| **Country** | **Correction** |
| --- | --- |
| **Qatar** | 3.84 |
| **US** | 1 |
| **Belgium** | 0.43 |

**Step 3. A multiplication factor was considered for inflation.**

**Table S6.** CHEERS Checklist.

The **ISPOR CHEERS Task Force Report**, *Consolidated Health Economic Evaluation Reporting Standards (CHEERS)—Explanation and Elaboration: A Report of the ISPOR Health Economic Evaluations Publication Guidelines Good Reporting Practices Task Force,* provides examples and further discussion of the 24-item CHEERS Checklist and the CHEERS Statement. It may be accessed via the *Value in Health* or via the ISPOR Health Economic Evaluation Publication Guidelines (17).

| **Section/Item** | **Item No.** | **Recommendations** |
| --- | --- | --- |
| **Title and abstract** | | |
| Title | 1 | Identify the study as an economic evaluation or use more specific terms such as “cost-effectiveness analysis”, and describe the interventions compared. |
| Abstract | 2 | Provide a structured summary of objectives, perspective, setting, methods (including study design and inputs), results (including base case and uncertainty analyses), and conclusions. |
| **Introduction** | | |
| Background and objectives | 3 | Provide an explicit statement of the broader context for the study. Present the study question and its relevance for health policy or practice decisions |
| **Methods** | | |
| Target population and subgroups | 4 | Describe characteristics of the base case population and subgroups analysed, including why they were chosen. |
| Setting and location | 5 | State relevant aspects of the system(s) in which the decision(s) need(s) to be made. |
| Study perspective | 6 | Describe the perspective of the study and relate this to the costs being evaluated. |
| Comparators | 7 | Describe the interventions or strategies being compared and state why they were chosen. |
| Time horizon | 8 | State the time horizon(s) over which costs and consequences are being evaluated and say why appropriate. |
| Discount rate | 9 | Report the choice of discount rate(s) used for costs and outcomes and say why appropriate. |
| Choice of health outcomes | 10 | Describe what outcomes were used as the measure(s) of benefit in the evaluation and their relevance for the type of analysis performed. |
| Measurement of effectiveness | 11a | *Single study-based estimates*: Describe fully the design features of the single effectiveness study and why the single study was a sufficient source of clinical effectiveness data. |
|  | 11b | *Synthesis-based estimates*: Describe fully the methods used for identification of included studies and synthesis of clinical effectiveness data. |
| Measurement and valuation of preference based outcomes | 12 | If applicable, describe the population and methods used to elicit preferences for outcomes. |
| Estimating resources and costs | 13a | *Single study-based economic evaluation*: Describe approaches used to estimate resource use associated with the alternative interventions. Describe primary or secondary research methods for valuing each resource item in terms of its unit cost.  Describe any adjustments made to approximate to opportunity costs |
|  | 13b | *Model-based economic evaluation*: Describe approaches and data sources used to estimate resource use associated with model health states. Describe primary or secondary research methods for valuing each resource item in terms of its unit cost. Describe any adjustments made to approximate to opportunity costs. |
| Currency, price date, and conversion | 14 | Report the dates of the estimated resource quantities and unit costs. Describe methods for adjusting estimated unit costs to the year of reported costs if necessary. Describe methods for converting costs into a common currency base and the exchange rate. |
| Choice of model | 15 | Describe and give reasons for the specific type of decision-analytical model used. Providing a figure to show model structure is strongly recommended. |
| Assumptions | 16 | Describe all structural or other assumptions underpinning the decision-analytical model. |
| Analytical methods | 17 | Describe all analytical methods supporting the evaluation. This could include methods for dealing with skewed, missing, or censored data; extrapolation methods; methods for pooling data; approaches to validate or make adjustments (such as half cycle corrections) to a model; and methods for handling population heterogeneity and uncertainty. |
| **Results** | | |
| Study parameters | 18 | Report the values, ranges, references, and, if used, probability distributions for all parameters. Report reasons or sources for distributions used to represent uncertainty where appropriate. Providing a table to show the input values is strongly recommended. |
| Incremental costs and outcomes | 19 | For each intervention, report mean values for the main categories of estimated costs and outcomes of interest, as well as mean differences between the comparator groups. If applicable, report incremental cost-effectiveness ratios. |
| Characterising uncertainty | 20a | *Single study-based economic evaluation*: Describe the effects of sampling uncertainty for the estimated incremental cost and incremental effectiveness parameters, together with the impact |
|  | 20b | *Model-based economic evaluation*: Describe the effects on the results of uncertainty for all input parameters, and uncertainty related to the structure of the model and assumptions. |
| Characterising heterogeneity | 21 | If applicable, report differences in costs, outcomes, or cost- effectiveness that can be explained by variations between subgroups of patients with different baseline characteristics or other observed variability in effects that are not reducible by more information. |
| **Discussion** | | |
| Study findings, limitations,  generalisability, and current knowledge | 22 | Summarise key study findings and describe how they support the conclusions reached. Discuss limitations and the generalisability of the findings and how the findings fit with current knowledge. |
| **Other** | | |
| Source of funding | 23 | Describe how the study was funded and the role of the funder in the identification, design, conduct, and reporting of the analysis. Describe other non-monetary sources of support. |
| Conflicts of interest | 24 | Describe any potential for conflict of interest of study contributors in accordance with journal policy. In the absence of a journal policy, we recommend authors comply with International Committee of Medical Journal Editors recommendations. |

**Table S7.** Validation tools

7.1 Assessment of the Validation Status of Health-Economic decision models (18)

| Question |  |
| --- | --- |
| Part A: Validation of the conceptual model |  |
| A1/ Face validity testing (conceptual model): Have experts been asked to judge the appropriateness of the conceptual model? | ✓ (DB) |
| A2/ Cross validity testing (conceptual model): Has this model been compared to other conceptual models found in the literature or clinical textbooks? | ✓  Our results were compared to previous studies as reported in the discussion section of the manuscript |
| Part B: Input data validation |  |
| B1/ Face validity testing (input data): Have experts been asked to judge the appropriateness of the input data? | ✓ (DB) |
| B2/ Model fit testing: When input parameters are based on regression models, have statistical tests been performed? | N/A |
| Part C: Validation of the computerized model |  |
| C1/ External review: Has the computerized model been examined by modelling experts? | ✓ (DB) |
| C2/ Extreme value testing: Has the model been run for specific, extreme sets of parameter values in order to detect any coding errors? | ✓ |
| C3/ Testing of traces: Have patients been tracked through the model to determine whether its logic is correct? | ✓ |
| C4/ Unit testing: Have individual sub-modules of the computerized model been tested? | ✓ |
| Part D: Operational validation |  |
| D1/ Face validity testing (model outcomes): Have experts been asked to judge the appropriateness of the model outcomes? | ✓ (DB) |
| D2/ Cross validation testing (model outcomes): Have the model outcomes been compared to the outcomes of other models that address similar problems? | Our results were compared to previous studies as reported in the discussion section of the manuscript |
| D3/ Validation against outcomes using alternative input data: Have the mod | ✓ |
| D4/ Validation against empirical data: Have the model outcomes been compared to empirical data? | ✓ |
| Part E: Other validation techniques |  |
| E1/ Other validation techniques: Have any other validation techniques been performed? | TECH-VER tool |

7.2 TECHnical VERification (TECH-VER) tool (19)

| Test description | Expected result | Result |
| --- | --- | --- |
| Model input (pre-analysis) calculations | | |
| Does the technology (drug/device, etc.) acquisition cost increase with higher prices? | N/A | Yes. Tested in the model |
| Does the probability of an event, derived from an OR/RR/HR and baseline probability, increase with higher OR/RR/HR? | Yes | Yes. Tested in the model |
| Additional check not in TECH-VER: Do the survival model predictions in Excel match those obtained from R? | N/A | N/A |
| Event/state calculations | | |
| The sum of the number of individuals at each health state should add up to the cohort size | Yes | Yes. Calculated in the trace |
| Check if all probabilities and number of individuals in a state are greater than or equal to 0 | Yes | Yes. Calculated in the trace |
| Check if all probabilities are smaller than or equal to 1 | Yes | Yes. Observed in the trace |
| Are the number of dead individuals in the previous period smaller than the number of dead individuals in the subsequent period | Yes | Yes. Calculated in the trace |
| In case of lifetime horizon, check if all individuals are dead at the end of the time horizon | Yes | Yes. Calculated in the trace |
| Are the QALYs equal to the life years if the utilities are set to 1? | Yes | Yes. Tested by placing utilities of 1 in the model |
| Are the QALYs equal to zero if the utilities are set to zero? | Yes | Yes. Tested by placing utilities of 0 in the model |
| If state utilities are lower, are QALYs lower? | Yes | Yes. Tested by placing the utility related to events to a lower value |
| Are costs zero if all costs are set to zero? | Yes | Yes. Tested by placing zero to all costs |
| If mortality risk is set to zero, do individuals die? | Yes | Yes. Tested by placing zero to all mortality inputs |
| If mortality risk is set to 1, do all individuals die in the first cycle? | Yes | Yes. Tested by changing the transition probability to death in cycle 1 to 1 |
| If all decision options have the same effectiveness, are life years and QALYs the same? | Yes | Yes. Tested in the model |
| If all decision options have the same effectiveness and costs, are all results the same? | Yes | Yes. Tested in the model |
| Is the number of individuals alive in the model, the same or lower as in the general population? | Yes | Yes |
| Is the QALY at each cycle, the same or lower than the general population? | Yes |  |
| If the inflation rate is higher, are the costs which are based on a reference from previous years higher too? | Yes | Yes. Tested by changing the inflation rate |
| Is the sum of all ingoing and outgoing transition probabilities in a state in a given cycle the same as the change in number of individuals? | Yes | Yes. Tested in the model |
| Are the number of individuals entering a tunnel state the same as the number of individuals leaving the tunnel state? | Yes | Yes. Tested in the trace |
| If the treatment acquisition cost is greater, are the costs greater? | Yes | Yes. Tested by increasing the costs of inputs |
| Are the time conversions for probabilities conducted correctly? | N/A | We used 6-month cycles |
| Result calculations | | |
| Do the more effective decision options yield greater QALYs and life years? | Yes | Yes. Tested in the sensitivity analysis |
| Do the more costly decision options yield greater treatment costs? | Yes | Yes. Tested in the sensitivity and scenario analyses |
| Are the total life years greater than the total QALYs? | Yes | Yes. As found in the results |
| Are the undiscounted results greater than the discounted results? | Yes | Yes. As found in the results |
| Is the ratio of the undiscounted total QALYs to the undiscounted total life years within the max and min of the utility inputs? | Yes | Yes. Tested in the model |
| Subgroup analysis results: Do subgroups with better baseline health have better outcomes? | N/A | N/A |
| Do the disaggregated results sum to the total results? | N/A | N/A |
| Are the life years with half-cycle correction lower than the life years without? | N/A | N/A |
| Are the discounted results equal to undiscounted if the discount rate is set to zero? | No | The undiscounted results are greater than the discounted results |
| If discount rates are higher, are the discounted results smaller? | Same | Same |
| Is the ratio of the total undiscounted treatment cost to the average duration of treatment similar to the treatment-related unit acquisition cost? | N/A | N/A |
| If the effect of the decision option is doubled, is the incremental effect approximately doubled? | Yes | Yes. Tested in the model |
| *Uncertainty analysis calculations* | | |
| Are all necessary parameters subject to uncertainty included in the OWSA? | Yes | Yes. Tested in the model |
| Does the OWSA include any parameters associated with joint uncertainty? | Yes |  |
| Are the upper and lower bounds used in the one-way sensitivity analysis using confidence intervals based on the statistical distribution assumed for that parameter? | Yes |  |
| Are the resulting ICER, incremental costs/QALYs with upper and lower bound of a parameter plausible and in line with a priori expectations? | Yes |  |
| Do all parameters used in the sensitivity analysis have appropriate associated distributions – upper and lower bounds should surround the deterministic value (i.e. upper bound ≥ mean ≥ lower bound) | Yes |  |
| Standard error and not standard deviation used in sampling | Yes | Yes, checked |
| Lognormal/gamma distribution for HRs and costs/resource use | Yes | Yes, checked |
| Beta for utilities and proportions/probabilities | Yes | Yes |
| Dirichlet for multinomial | N/A | N/A |
| Multivariate normal for correlated inputs | N/A | N/A |
| Normal for other variables as long as samples do not violate the requirement to remain positive when appropriate | N/A | N/A |
| Check PSA output mean costs, QALYs, and ICER compared with the deterministic results. Is there a large discrepancy? | No | No. As reported in the results |
| If you take new PSA runs from the Microsoft Excel model do you get similar results? | Yes | Yes |
| Is(are) the CEAC line(Overall validation) in line with the CE scatter plots and the efficient frontier? | Yes | Yes |
| Does the PSA cloud demonstrate an unexpected behaviour or have an unusual shape? | No | No |
| Is the sum of all CEAC lines equal to 1 for all WTP values? | N/A | N/A |
| Do the explored scenario analyses provide a balanced view on the structural uncertainty (i.e. not always looking at more optimistic scenarios)? | Yes | Yes |
| Are the scenario analysis results plausible and in line with a priori expectations? | Yes | Yes |
| Check the correlation between two PSA results (i.e. costs/QALYs under the SoC and costs/QALYs under the comparator). Should be very low (very high) if different (same) random streams are used for different arms | Yes | Yes, checked |
| If a certain seed is used for random number generation (or previously generated random numbers are used), check if they are scattered evenly between 0 and 1 when they are plotted | N/A | N/A |
| Is the mean of the parameter samples generated by the model similar to the point estimate for that parameter? Use graphical methods to examine distributions, functions | N/A | N/A |
| Do sensitivity analyses include any parameters associated with methodological/structural uncertainty? | Yes | Yes |
| Value of information analysis if applicable: Was this implemented correctly? | N/A | N/A |
| Which types of analysis? | N/A | N/A |
| Is EVPI larger than all individual EVPPIs? | N/A | N/A |
| Is EVPPI for a (group of) parameters larger than the EVSI of that (group) of parameter(s)? | N/A |  |
| Are the results from EVPPI in line with OWSA or other parameter importance analysis (e.g. ANCOVA)? | N/A |  |
| Did the electronic model pass the black-box tests of the previous verification stages in all PSA iterations and in all scenario analysis settings? | N/A | N/A |
| Check if all sampled input parameters in the PSA are correctly linked to the corresponding event/state calculations | Yes | Yes. Checked |
| Overall validation/other supplementary checks | | |
| Compare the model outcomes with clinical inputs used in the model, findings from the literature, clinical expert knowledge and other model outcomes | Yes | The results were compared with international sources |

*Advishe: Assessment of the Validation Status of Health-Economic decision models, CEAC: Cost-Effectiveness Acceptability Curve, CPRD: Clinical Practice Research Datalink, CTTC: Cholesterol Treatment Trialists’ Collaboration. CV: Cardiovascular. EVPI: Expected Value of Perfect Information, EVPPI: Expected Value of Perfect Parameter Information, EVSI: Expected Value of Sample Information, HR: Hazard Ratio, ICER: Incremental Cost-Effectiveness Ratio, LLT: Lipid Lowering Treatment, OR: Odds Ratio, OWSA: One-Way Sensitivity Analysis, PSA: Probabilistic Sensitivity Analysis. QALYs: Quality-Adjusted Life Years, RR: Risk Ratio. SoC: Standard of Care, WTP: Willingness To Pay

**References**

1. Bradley P, Shiekh M, Mehra V, Vrbicky K, Layle S, Olson MC, et al. Improved efficacy with targeted pharmacogenetic-guided treatment of patients with depression and anxiety: A randomized clinical trial demonstrating clinical utility. J Psychiatr Res. 2018 Jan;96:100–7.

2. Jithesh PV, Abuhaliqa M, Syed N, Ahmed I, El Anbari M, Bastaki K, et al. A population study of clinically actionable genetic variation affecting drug response from the Middle East. npj Genomic Med [Internet]. 2022;7(1):10. Available from: https://doi.org/10.1038/s41525-022-00281-5

3. Rush AJ, Trivedi MH, Wisniewski SR, Nierenberg AA, Stewart JW, Warden D, et al. Acute and longer-term outcomes in depressed outpatients requiring one or several treatment steps: a STAR*D report. Am J Psychiatry. 2006 Nov;163(11):1905–17.

4. Sim K, Lau WK, Sim J, Sum MY, Baldessarini RJ. Prevention of Relapse and Recurrence in Adults with Major Depressive Disorder: Systematic Review and Meta-Analyses of Controlled Trials. Int J Neuropsychopharmacol. 2015 Jul;19(2).

5. Sluiter RL, Janzing JGE, van der Wilt GJ, Kievit W, Teichert M. An economic model of the cost-utility of pre-emptive genetic testing to support pharmacotherapy in patients with major depression in primary care. Pharmacogenomics J [Internet]. 2019;19(5):480–9. Available from: https://doi.org/10.1038/s41397-019-0070-8

6. Carta A, Del Zompo M, Meloni A, Mola F, Paribello P, Pinna F, et al. Cost-Utility Analysis of Pharmacogenetic Testing Based on CYP2C19 or CYP2D6 in Major Depressive Disorder: Assessing the Drivers of Different Cost-Effectiveness Levels from an Italian Societal Perspective. Clin Drug Investig. 2022 Sep;42(9):733–46.

7. Fabbri C, Kasper S, Zohar J, Souery D, Montgomery S, Albani D, et al. Cost-effectiveness of genetic and clinical predictors for choosing combined psychotherapy and pharmacotherapy in major depression. J Affect Disord. 2021 Jan;279:722–9.

8. Shams MEE, Arneth B, Hiemke C, Dragicevic A, Müller MJ, Kaiser R, et al. CYP2D6 polymorphism and clinical effect of the antidepressant venlafaxine. J Clin Pharm Ther. 2006 Oct;31(5):493–502.

9. Sullivan PW, Ghushchyan V. Preference-Based EQ-5D index scores for chronic conditions in the United States. Med Decis Mak an Int J Soc Med Decis Mak. 2006;26(4):410–20.

10. Kuyken W, Byford S, Taylor RS, Watkins E, Holden E, White K, et al. Mindfulness-based cognitive therapy to prevent relapse in recurrent depression. J Consult Clin Psychol. 2008 Dec;76(6):966–78.

11. Births & Deaths in the State of Qatar. 2019. Planning and statistics authority [Internet]. [cited 2024 March 12]. Available from: https://www.psa.gov.qa/en/statistics/Statistical%20Releases/Population/BirthsDeaths/2019/Birth_death_review_2019_EN.pdf.

12. Bousman CA, Stevenson JM, Ramsey LB, Sangkuhl K, Hicks JK, Strawn JR, et al. Clinical Pharmacogenetics Implementation Consortium (CPIC) Guideline for CYP2D6, CYP2C19, CYP2B6, SLC6A4, and HTR2A Genotypes and Serotonin Reuptake Inhibitor Antidepressants. Clin Pharmacol Ther. 2023 Jul;114(1):51–68.

13. Elbakary N, Ouanes S, Riaz S, Abdallah O, Mahran I, Al-Khuzaei N, et al. Prevalence, median time, and associated factors with the likelihood of initial antidepressant change: a cross-sectional study in Qatar. BMC Psychiatry. 2021 Feb;21(1):115.

14. Briggs A, Claxton K, Sculpher M. Decision Modeling for Health Economics Evaluation. Briggs A, Claxton K, Sculpher M, Eds. Oxford, Oxford University. Press, 2006.

15. Qatar. 2021. Institute for Health Metrics and Evaluation [Internet]. [Cited 2024 March 12]. Available from: https://www.healthdata.org/research-analysis/health-by-location/profiles/qatar.

16. Births & Deaths In the State of Qatar. 2016. Ministry of Development Planning and Statistics [Internet]. [Cited 2024 March 12]. Available from: https://www.psa.gov.qa/en/statistics/Statistical%20Releases/Population/BirthsDeaths/2016/Birth_death_2016_EN.pdf.

17. Husereau D, Drummond M, Augustovski F, de Bekker-Grob E, Briggs AH, Carswell C, et al. Consolidated Health Economic Evaluation Reporting Standards 2022 (CHEERS 2022) Statement: Updated Reporting Guidance for Health Economic Evaluations. Value Heal J Int Soc Pharmacoeconomics Outcomes Res. 2022 Jan;25(1):3–9.

18. Vemer P, Corro Ramos I, van Voorn GAK, Al MJ, Feenstra TL. AdViSHE: A Validation-Assessment Tool of Health-Economic Models for Decision Makers and Model Users. Pharmacoeconomics. 2016 Apr;34(4):349–61.

19. Büyükkaramikli NC, Rutten-van Mölken MPMH, Severens JL, Al M. TECH-VER: A Verification Checklist to Reduce Errors in Models and Improve Their Credibility. Pharmacoeconomics. 2019 Nov;37(11):1391–408.
